# Povetacicept (ALPN-303; TACI vTD-Fc), an enhanced, potent dual inhibitor of BAFF and APRIL, ameliorates experimental autoimmune myasthenia gravis in C57BL/6N mice

**DOI:** 10.3389/fimmu.2025.1533093

**Published:** 2025-06-06

**Authors:** Elena Rinaldi, Elisa Puleo, Alessandra Consonni, Martina Miglietti, Renato Mantegazza, NingXin Wang, Stanford L. Peng, Katherine E. Lewis, Stacey R. Dillon, Fulvio Baggi

**Affiliations:** ^1^ Department of Clinical Neurosciences, Fondazione Istituto di Ricovero e Cura a Carattere Scientifico (IRCCS) Istituto Neurologico Carlo Besta, Milan, Italy; ^2^ Bioanalytical Sciences, Alpine Immune Sciences, a Vertex Company, Seattle, WA, United States; ^3^ Research, Alpine Immune Sciences, a Vertex Company, Seattle, WA, United States

**Keywords:** BAFF - B-cell activating factor, APRIL (TNFSF13), myasthenia gravis, experimental autoimmune myasthenia gravis, animal model, TACI (TNFRSF13B), B cell

## Abstract

**Background and Objectives:**

Myasthenia gravis (MG) is a T cell-dependent, B cell-mediated autoimmune disease targeting the acetylcholine receptor (AChR) and other proteins of the neuromuscular junction postsynaptic membrane. Production of pathogenic autoantibodies results from B cell activation and expansion of antibody-secreting cells, including plasma cells, whose differentiation and survival are reliant on the TNF family cytokines APRIL and BAFF. Povetacicept (ALPN-303; TACI vTD-Fc) is an Fc fusion protein of an engineered TACI domain with significantly more potent dual inhibition of APRIL and BAFF than wild-type (WT) TACI-Fc (e.g., telitacicept).

**Methods:**

In this study, the activity of povetacicept was evaluated in the mouse experimental autoimmune MG (EAMG) model, compared to (i) telitacicept, (ii) a depleting anti-CD20 antibody, (iii) neonatal Fc receptor blocker efgartigimod, (iv) a matched Fc control protein, and (v) PBS as vehicle.

**Results:**

Therapeutic administration of povetacicept ameliorated clinical manifestations in EAMG mice and was associated with significantly lower levels of immunoglobulin subclasses and anti-AChR antibody titers in serum, along with increased muscle AChR content – superior to the evaluated comparators. Povetacicept treatment also reduced the number of total B220^+^ and Ki67^+^ proliferating cells in draining lymph node follicles and resulted in modifications of splenic T and B cell subset frequencies, compared to controls.

**Discussion:**

The potent, dual BAFF/APRIL inhibitor povetacicept significantly improves clinical disease activity in EAMG, associated with reductions in pathogenic anti-AChR autoantibodies and superior to comparator therapeutic interventions based on WT TACI-Fc, CD20 depletion, or FcRn inhibition. Povetacicept may therefore confer beneficial clinical outcomes in the treatment of MG and other autoantibody-related neurological diseases.

## Introduction

Myasthenia gravis (MG) is a chronic T cell-dependent, B cell-mediated autoimmune disease of the neuromuscular junction (NMJ), characterized by fluctuating levels of muscle weakness and fatigue with a rapid onset and worsening during normal daily activity or after repeated muscle work (1). In 80-85% of MG patients, pathogenic autoantibodies (autoAbs) are directed against the nicotinic acetylcholine receptor (AChR) located at the top of the post-synaptic membrane clefts (1); anti-AChR autoAbs belong to the IgG1 and IgG3 subtypes, which are able to activate complement components and are responsible for the impaired conduction of the contraction signal from motoneurons to the muscle fiber (2). A minority of MG patients have antibodies against muscle-specific kinase (MuSK) or lipoprotein-receptor-related protein 4 (LRP4); in a small portion of MG patients, serum autoAbs are not detected, and these patients are classified as triple seronegative MG (1).

Current treatments for MG include drugs that target the disease symptoms, e.g., aiming to counteract the muscle fatigability using acetylcholinesterase inhibitors, and combinations of medications with steroids or other immunosuppressant agents, such as azathioprine, mycophenolate, or methotrexate (3). While the majority of MG patients respond to these treatments, 10–15% remain refractory or experience severe adverse events in response to treatments, thus highlighting the need for more targeted, effective, and safer therapies. In recent years, new treatments have been developed that are designed to interfere with the complement system or inhibit the recycling of immunoglobulin (Ig)G via the neonatal Fc receptor (FcRn), as recently reviewed (3). In addition, targeted B cell therapies (e.g., anti-CD20) have been evaluated in clinical trials, and have demonstrated beneficial effects, further confirming the importance of B cells in pathogenesis of MG (4).

B cells play important roles in MG, including autoAb production, complement activation, and cytokine release (5, 6). Among the key B cell related cytokines identified in MG, both B-cell Activating Factor (BAFF) and A PRoliferation-Inducing Ligand (APRIL), members of the tumor necrosis factor (TNF) superfamily (7, 8), have been found to be upregulated in MG patients compared to healthy controls (9, 10). Furthermore, it has been observed that BAFF-receptor (BAFF-R) expression in peripheral blood B cells is significantly higher in patients with refractory MG compared with those with nonrefractory MG (11). MG patients also have enhanced B-cell activation signals, particularly those with thymic follicular hyperplasia in which the presence of germinal centers (GC) could provide signals to promote AChR-specific B-cell survival and activation (12).

BAFF binds with varying affinity to B cell-expressed BAFF-R, Transmembrane Activator and CAML Interactor (TACI), and B-Cell Maturation Antigen (BCMA), while APRIL binds TACI and BCMA and heparan sulfate proteoglycans like CD138 on plasma cells. TACI and BCMA are particularly expressed on activated mature B cells, memory B cells, and plasma cells (13), whereas BAFF-R expression is highest on B cells earlier in the differentiation pathway (14). Interactions between APRIL and BAFF and their receptors activate a series of signal cascades that lead to increased presentation of antigens by B cells, co-stimulation of B cell proliferation, increased cell survival, regulation of B-cell immunological tolerance, and Ig class switch recombination.

Clinical exploration of BAFF/APRIL inhibition in MG has been initiated. Telitacicept has been granted orphan drug designation by the U.S. Food and Drug Administration (FDA) as a potential treatment for MG and has exhibited some promising preliminary data in an uncontrolled clinical study in China. In the open label Phase 2 trial in China, nearly six months of treatment with telitacicept lessened disease severity in adults with AChR^+^ autoAb MG, but its efficacy seemed somewhat limited and lacked evidence of long-term remission; results from a randomized study will be important to confirm these early results (15, 16).

Povetacicept is a novel variant of TACI‐Fc with enhanced binding for both APRIL and BAFF and can inhibit both cytokines more potently than WT TACI‐Fc proteins (15). Povetacicept comprises the cysteine-rich-2 (CRD2) domain of the extracellular portion of the human TACI receptor containing 3 amino acid substitutions that differ from the WT CRD2 domain, fused to a modified human IgG1 Fc domain that lacks effector function (15, 16). The WT TACI-Fc proteins telitacicept and atacicept comprise the full-length (CRD1+CRD2) TACI domains fused to an inert human IgG1-derived Fc domain (17). Povetacicept was well tolerated in a first-in-human study in healthy volunteers (NCT05034484) and exhibited dose-related pharmacokinetics and expected pharmacodynamic effects, including dose-related reductions in serum IgA, IgM, and IgG, and in circulating antibody-secreting cells (18). No prior studies have investigated BAFF/APRIL inhibition in the experimental model of autoimmune myasthenia gravis (EAMG), a well-characterized model used to study the mechanisms involved in MG (19, 20), but exploration of the potential for BAFF/APRIL in MG patients may be enhanced by a comparative study in EAMG vs. other mechanisms of clinical interest. In the current study, we have evaluated the effects of povetacicept administration in mouse EAMG and compared the effects to those of a WT TACI-Fc protein, an anti-CD20 antibody, and an FcRn inhibitor (21, 22).

## Materials and methods

### Animals

Female C57BL/6N mice, 6–8 weeks of age, were purchased from Charles River Laboratories Italia (Calco, Italy) and housed at the animal facility of the Foundation IRCCS Neurological Institute Carlo Besta (Milan, Italy). Animals were housed in groups of 3–5 mice per cage, with artificial circadian light cycle (12-h light/12-h dark), at a temperature of 23°C, and a standard chow diet and water provided ad libitum. All procedures involving animals were approved by the Institute Ethical Board and Italian Ministry of Health (452/2022-PR) and were performed according to the Italian Principle of Laboratory Animal Care (DDL 116/92 and DLgs 26/2014) and the European Communities Council Directive 86/609/EEC and 2010/63/UE.

### Purification of torpedo AChR

Torpedo AChR (TAChR) was purified from *Torpedo californica* electric organ (Aquatic Research Consultants, San Pedro, CA), according to the alkali-stripped membrane protocol (23, 24), with minor modifications. All steps were performed on ice. Briefly, the electric organ was homogenized in 10 mM sodium phosphate buffer, 1 mM EDTA, 0.02% NaN_3_, 0.01 mM PMSF, 0.1 M NaCl (pH 7.8) for 4 min, high speed, followed by 30 sec periods at 25,000 rpm using a Ystral X1020 Ultra-turrax, and then centrifuged for 1 h at 50,000 x *g*. The pellet was resuspended in phosphate buffer with 30% sucrose (w/w) and layered onto a discontinuous sucrose gradient consisting of 2 ml of 50% (w/w), 3 ml of 39% (w/w) and 4 ml of 35% (w/w) sucrose. Tubes were centrifuged at 100,000 x *g* for 1 h, 4°C. After centrifugation, the middle light-scattering band was collected, diluted 2-fold into sodium phosphate buffer, and centrifuged for 1 h at 90,000 x *g*, 4°C. The pellet was resuspended in ice-cold distilled water, the pH adjusted to 11.0 with NaOH and immediately centrifuged for 30 min at 90,000 x *g*. The TAChR was then solubilized from membranes with 2% sodium deoxycholate, overnight at 4°C, then centrifuged at 50,000 x *g* for 1 h, 4°C. Sodium deoxycholate was partially removed by progressive dialysis (1%, 0.5%, and then 0.05% in sodium phosphate buffer). Aliquots of TAChR were stored at –80°C. The concentration of TAChR was determined as α-bungarotoxin (BuTX)-binding sites/ml; protein concentration was assessed by the BCA Protein Assay Kit (Thermo Scientific). TAChR specific activity was equal to 0.87–1.1 nmol of [^125^I]-labelled-αBuTX-binding sites/mg, in line with literature data (24).

### Experimental autoimmune myasthenia gravis

EAMG was induced in mice according to a consensus guideline (19) by subcutaneous immunization in the hind limbs (multiple sites) with 20 µg of purified TAChR from *Torpedo californica* electric organ, emulsified in complete Freund’s adjuvant (CFA; Difco, 1:1 ratio), in a total volume of 200 µl. After the first immunization, two subsequent TAChR boosts (20 µg of TAChR emulsified in incomplete Freund’s adjuvant [IFA], 200 µl/mouse) were administered, 4 and 8 weeks post-immunization to induce EAMG (**Supplementary Figure 1**). Each animal was weighed and scored at the beginning of each experiment, twice a week until the second boost, and then every other day (or twice a day if the animal demonstrated severe weakness) by blinded investigators, unaware of the treatments (19), until termination 13 weeks following the initial immunization. Clinical scores of disease were graded as follows: grade 0, normal strength and no muscle weakness, even after exercise (i.e., 30 consecutive paw grips to a steel grid cage top); grade 1, normal at rest but fatigability or weakness (tremor or inability to raise head or hunched posture) observed after exercise; grade 2, clinical signs of weakness present at rest; grade 3, severe clinical signs of weakness: i.e., no ability to grip, hindlimb paralysis, respiratory distress/apnoea, weight loss >15%; grade 4, humane endpoint/sacrifice due to very severe clinical signs of weakness. Weak mice after exercise that recovered quickly (i.e., within 30 sec) were graded 0.5. Disease was confirmed by intraperitoneal (i.p.) injections of 0.75 mg of neostigmine (an anti-cholinesterase inhibitor), which results in temporary reversal of clinical signs (20). A low level of anesthesia (2% isoflurane in 60:40 N_2_O:O_2_; flow rate 0.8 l/min) was administered to animals prior to immunizations and treatments. Animals were sacrificed via inhalation of carbon dioxide (CO_2_ euthanasia); blood, spleens, draining lymph nodes, and carcasses were collected for endpoint analyses.

### Test articles and dosing

Povetacicept (15), the matched Fc control recombinant protein, telitacicept (WT TACI-Fc; sourced from Clinigen), efgartigimod (sourced from Clinigen), and anti-mouse CD20 monoclonal antibody (mAb) (Ultra-LEAF™ Purified anti-mouse CD20 mAb; clone SA271G2, rat IgG2b, BioLegend) were provided by Alpine Immune Sciences (Seattle, WA). Povetacicept (~10 mg/kg per dose) or molar-matched levels of telitacicept (~12 mg/kg) or Fc control (~8.3 mg/kg) were administered twice weekly. Efgartigimod (~20 mg/kg) or sterile PBS as vehicle (PBS-EAMG) were also administered twice weekly; anti-CD20 was administered once weekly (0.25 mg/dose, as recommended by the manufacturer and confirmed to deplete B cells in mice). Test articles were resuspended in sterile PBS and administered by i.p. injection (100 µl). The human Fc control protein was matched to the Fc domain in povetacicept and lacks effector function, as it does not bind Fc receptors (FcRs) other than the neonatal FcR (FcRn), nor does it fix complement (15, 16). Three independent experiments were performed as outlined in **Supplementary Figure 1**: a pilot experiment, in which EAMG animals (n=15, randomized into three groups of 5 mice each) were treated i.p. with povetacicept beginning either from Week 7 or Week 9 (i.e., one week before, or one week after the second AChR/IFA boost, respectively), or with PBS, to perform an initial assessment of the treatment protocol; then two comparator treatment experiments were executed in which dosing began one week after the second AChR/IFA boost. In the first comparator experiment, EAMG mice (n=48, randomized in four groups of 12 mice each) received povetacicept, telitacicept, Fc control recombinant protein, or PBS, and in the second experiment, EAMG mice (n=60, randomized into five groups of 12 mice each) received povetacicept, Fc control, efgartigimod, anti-CD20, or PBS. Sample size was determined according to guidelines for preclinical research in animals and specifically in the mouse EAMG model (19, 25).

### Determination of muscle AChR content

The amount of AChR in muscles from EAMG mice was assayed by radioimmunoprecipitation (20), with minor modifications. Briefly, whole carcasses were weighed and homogenized for 1 min at high speed in four volumes of homogenization buffer (0.1 M NaCl, 10 mM NaN_3_, 0.01 M EDTA, 0.01 M EGTA, 0.01 M iodoacetamide, 1 mM PMSF, 1 mM sodium phosphate buffer, pH 7.5). The homogenized extract was centrifuged 30 min at 15,000 rpm (4°C) and pellets were further homogenized for 1 min at high speed in one volume of homogenization buffer. Triton X-100 was added at 10% concentration to solubilize AChR from membranes. Extracts were kept for 4 h, 4°C, on a shaker, then centrifuged at 15,000 rpm for 30 min (4°C). Supernatants were collected and centrifuged for 30 min at 26,000 rpm (4°C) after which any fat on the surface was discarded. Muscle AChR content was quantified by immunoprecipitation assay. Briefly, duplicate aliquots of 0.1 ml of mouse muscle AChR crude extract were incubated with 0.2 pM [^125^I]α-BuTX. High-titer serum from EAMG mice was then added to each sample and incubated overnight (4°C). Complexes were precipitated by adding an excess of rabbit anti-mouse IgG (Sigma) and centrifuging samples for 10 min, 3000 rpm at 4°C. Pellets were washed twice in wash buffer (100 mM phosphate buffer pH 7.4, 1.4 mM NaCl, 4 mM KCl, 0.5% Triton X-100). [^125^I]-αBuTX labelling was assessed using a gamma-counter (PerkinElmer). Results were expressed as femtomoles of toxin-binding sites/gram of carcass; non-specific binding from extracts pre-incubated with unlabelled α-BuTX was subtracted from each sample.

### Serum anti-AChR antibody measurement

Anti-mouse AChR antibodies were measured in terminal serum samples by a radioimmunoprecipitation assay (20, 26), using AChR extracted from naïve mice labelled with 0.2 pmoles [^125^I] α-BuTX. Serum samples from experimental mice were incubated overnight with the labelled AChR. AChR-antibody complexes were precipitated by adding an excess of a secondary rabbit polyclonal anti-mouse IgG (Sigma) for 2.5 h, at room temperature (RT), followed by centrifugation for 10 min, 3000 rpm, 4°C. Pellets were washed twice in wash buffer (100 mM Phosphate buffer pH 7.4, 1.4 mM NaCl, 4 mM KCl, 0.5% Triton X-100). [^125^I]-αBuTX labelling was assessed using a gamma-counter (PerkinElmer). Serum samples incubated with an excess of unlabelled α-BuTX (Life Technologies) were subtracted from test samples. Anti-mouse AChR antibody titers were expressed as picomoles of [^125^I] α-BuTX-binding sites precipitated per milliliter of serum.

### Serum total immunoglobulin analysis

Serum samples, collected at Week 9 (just prior to the first dose) and at termination (Week 13) in each of the comparator treatment experiments, were analyzed for concentrations of total mouse IgM, IgG1, IgG2b, IgG3, and IgA using a magnetic bead multiplexed mouse Ig isotyping kit (Millipore-Sigma) per the manufacturer’s instructions.

### Serum exposure and anti-drug antibody measurements

In the first comparator experiment, serum samples were collected just prior to test article dosing at Week 9.6, 10.6, and 11.6, and at Week 12.6 and 13 (termination) for analysis of povetacicept and telitacicept concentrations using an enzyme-linked immunosorbent assay (ELISA) as described (15). Titers of anti-drug antibodies (ADA) in terminal serum samples of povetacicept- and telitacicept-treated mice were analyzed by measuring the optical density (OD) (at 450 nm) of horseradish peroxidase (HRP)-labelled Fc-specific, F(ab’)2 fragment goat anti-mouse IgG bound to a serum-test article complex, in which serial dilutions of serum were incubated in wells pre-coated with the appropriate test article. The OD data for each study sample was compared to a cut-point OD, which was the OD value measured in serum from naive C57BL/6 mice (n = 6), diluted 1:100. Interquartile range analysis was used for outlier determination in the naïve mouse group.

### Immunofluorescence analysis of lymph nodes

In the first comparator treatment experiment, draining (inguinal) lymph nodes (LNs) were collected from 6 mice, chosen at random, per treatment group and 3 naïve healthy donor (HD) female C57BL/6 mice for immunofluorescence analysis. To ensure that the immunofluorescence analysis performed in a subset of mice was representative of the entire set, EAMG scores from each group for all mice were compared to those of the subset selected for immunofluorescence analysis (**Supplementary Figure 2**). Following overnight fixation in 4% paraformaldehyde (4°C), the LNs were placed in 15% sucrose/PBS, followed by 30% sucrose/PBS, for cryopreservation; samples were then embedded in optimal cutting temperature (OCT) compound (Killik). Ten-μm serial cryosections were used for immunofluorescence staining. Briefly, following 1 h incubation with blocking buffer (5% BSA and 0.5% Tween in PBS), sections were incubated with primary antibodies (2 h, RT) and with appropriate secondary antibodies (1 h, RT); the antibodies used are listed in **Supplementary Table 1**. Nuclei were stained with 4′,6-diamidino-2-phenylindole (DAPI, Thermo Fisher). Slices were mounted in FluorSaveTM Reagent (Millipore) and at least 3 histological sections per lymph node acquired via confocal microscopy (C1/TE2000-E microscope; Nikon) using a 20x (numerical aperture 0.5) objective; all images were acquired with the following parameters: 2048x2048 pixel image resolution, z-stack 2 µm (at least 10 stacks), pinhole 30 µm, 568-PMT (red channel) gain=154, 488-PMT (green channel) gain=123. Maximum Intensity Z-projection was obtained from z stack-images, and green and red fluorescence channels were split and converted in greyscale; the total area of the image and the mean grey value were measured to calculate the integrated density for each marker/fluorophore. IntDen values, calculated by Fiji software as units/square micron, were then converted to units/square millimeter. Regions of interest (ROI), identifying B cell follicles, were tracked on the basis of B220^+^ (red) fluorescence and integrated density was again measured on 3–10 follicles per mouse; the same ROI was then used to measure the Ki67^+^ (green) integrated density to evaluate proliferating cells in follicles. All image analyses were performed using Fiji software (NIH, USA) (27).

### Flow cytometry analysis of splenocytes

Spleens were weighed and dissociated to single-cell suspensions by pressing through a 70 μm cell strainer, cells were collected in PBS and centrifuged; red blood cells were lysed by incubation with Ammonium-Chloride-Potassium buffer (ACK) for 5 min on ice. Spleen cells were counted by the Trypan Blue exclusion method on an hemacytometer, slowly frozen in 10% dimethyl sulfoxide (DMSO)/fetal bovine serum (FBS) and stored at 10x10^6^ cells/cryotube in liquid nitrogen for subsequent analyses. Prior to flow cytometry staining, frozen cell aliquots were thawed in a 37°C water bath and counted by Trypan Blue to assess viability; 1-2x10^6^ viable splenocytes (in PBS) were incubated with live-dead dye (eBioscience Fixable Viability Dye [FVD] eFluor™ 506, Invitrogen; 1:1000 in PBS); non-specific binding was prevented with mouse Fc receptor block (unlabelled CD16/CD32 mAb Clone 93, e-Bioscience; 0.5 μg/tube).

Splenic B cell lineage was defined as: B cells, CD19^+^B220^+^; germinal center B cells (B_GC_), CD19^+^B220^+^CD95^+^GL7^+^; plasma cells (PC), CD138^+^TACI^+^; long-lived PC (PC_LL_), CD138^+^TACI^+^CD19^-^B220^-^ (28). Splenic T cell lineage (CD4^+^ and CD8^+^) was identified as: effector memory CD4^+^ T cells (CD4_EM_), CD4^+^CD44^+^CD62L^-^; effector memory CD8^+^ T cells (CD8_EM_), CD8^+^CD44^+^CD62L^-^; naïve CD4^+^ T cells (CD4_naïve_), CD4^+^CD44^-^CD62L^+^; naïve CD8^+^ T cells (CD8_naïve_), CD8^+^CD44^-^CD62L^+^; central memory CD4^+^ T cells (CD4_CM_), CD4^+^CD44^+^CD62L^+^; central memory CD8^+^ T cells (CD8_CM_), CD8^+^CD44^+^CD62L^+^; follicular helper CD4^+^ T cells, CD4^+^PD1^+^CXCR5^+^; regulatory CD4^+^ T cells, CD4^+^CD25^+^FoxP3^+^. FoxP3/Transcription Factor Staining Buffer Set (eBioscience) was used for cell fixation/permeabilization; staining was performed at 4°C, 1 hour, in 5 mM EDTA, 5% BSA, PBS (staining buffer). Antibody-fluorophore combinations used for flow cytometry analysis are described in **Supplementary Table 1**.

All flow cytometry analyses were performed on gated viable (defined by FSC-A/SSC-A and by FVD staining) and single (defined by SSC-H/SSC-A) cells; gating strategies are reported in **Supplementary Figure 3**. Antibody concentrations were individually titrated beforehand to select optimal concentrations for each panel. Positive and negative gates were established using the fluorescence-minus-one (FMO) technique. Flow cytometry analyses were performed on an Attune NxT Flow Cytometer (Thermo Fisher). Data are reported as cell and cell subset percentages; it was not possible to accurately calculate total cell subset numbers per spleen since splenocytes were frozen after collection and once thawed, could therefore not be considered representative of the cellular composition of fresh spleen.

### Statistical analysis

The Mann-Whitney test was used for analysis of statistically significant differences between 2 groups (**Supplementary Figure 2**). For all other data sets (>2 groups), experimental data were analyzed via one-way analysis of variance (ANOVA), followed by Tukey’s multiple comparisons test, or via the Kruskal–Wallis test and Dunn’s multiple comparisons test for ordinal and non-normally distributed data; normality of data was determined via the Kolmogorov-Smirnov test. All p values were corrected for multiple comparisons; p < 0.05 was considered statistically significant. GraphPad Prism software (GraphPad Software, Inc.) was used for statistical analyses and graphical presentation of data.

## Results

### Povetacicept treatment ameliorates EAMG

**Figure 1:**
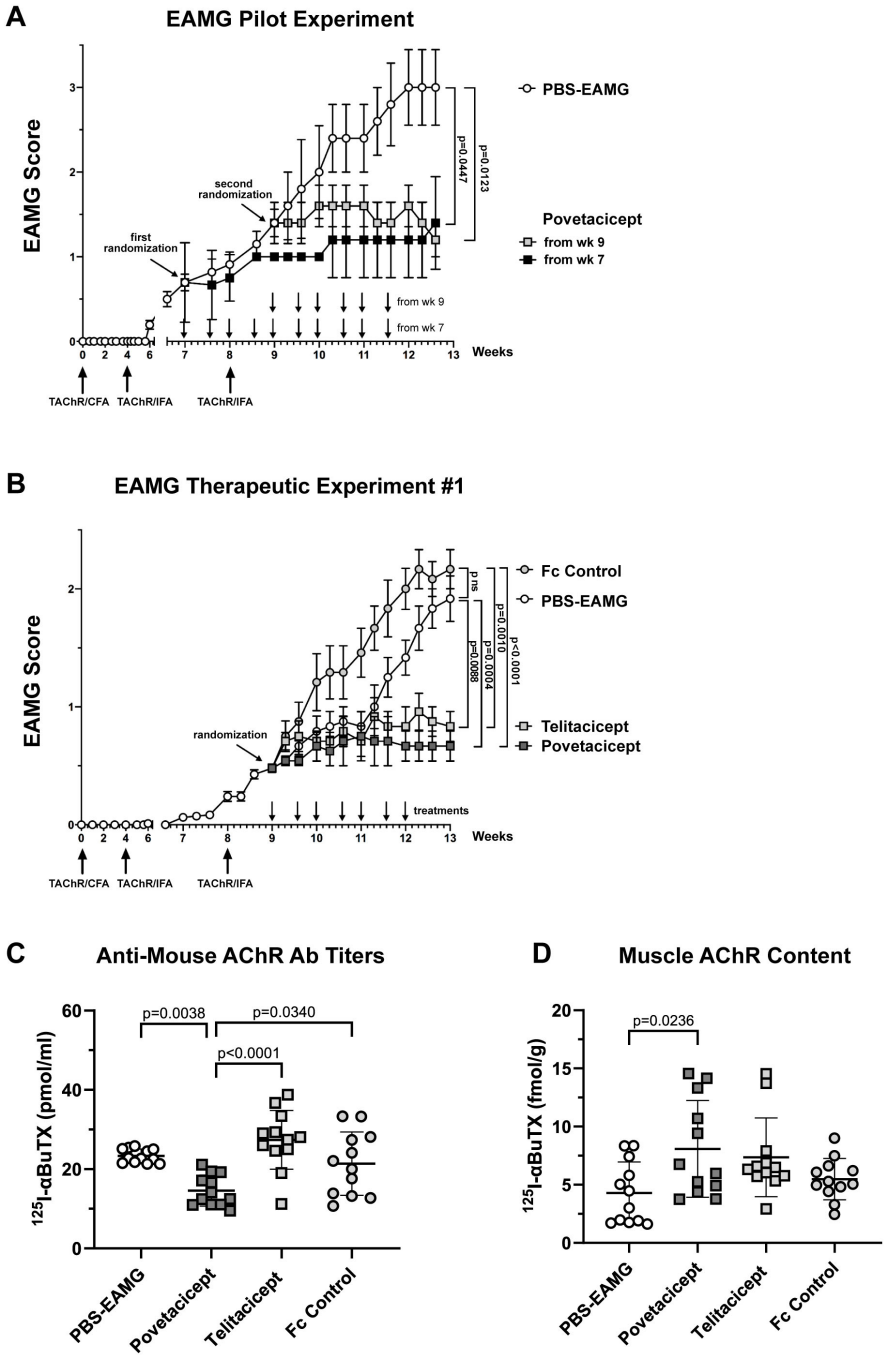
Administration of povetacicept reduces clinical signs of EAMG. **(A)** EAMG pilot experiment. Povetacicept was administered i.p. twice weekly from Week (wk) 7 (grey squares, n=5 mice, 10 doses total) or from Week 9 (black squares, n=5 mice, 6 doses total). EAMG control mice received vehicle (PBS) only, from Week 7 (white circles, n=5 mice, 10 doses total) (PBS-EAMG). TAChR immunization and boosts are indicated with upward arrows and i.p. treatments with downward arrows on the x-axis. **(B)** EAMG therapeutic experiment. EAMG mice received povetacicept (grey squares, n=12 mice, 7 doses total), telitacicept (light grey squares, n=12 mice, 7 doses total), an Fc control (light grey circles, n=12 mice, 7 doses total), or vehicle (PBS-EAMG) (white circles, n=12 mice, 7 doses total) via twice weekly i.p. injections. TAChR immunization and boosts are indicated with upward arrows and i.p. treatments with downward arrows on the x-axis. Statistical significance of clinical scores between groups in A and B was calculated with the Kruskal-Wallis non-parametric test with Dunn’s multiple comparisons test; data are presented as mean EAMG scores ± the standard error of the mean (SEM), with mice being scored for disease as described in the Methods section. **(C)** Anti-mouse AChR antibody titers in individual mice were measured by a radioimmunoprecipitation assay detailed in the Methods and expressed as pmoles of immunoprecipitated ^125^I-αBuTx binding sites per ml of serum; horizontal and vertical bars represent the mean and standard deviation (SD), respectively. **(D)** AChR muscle content in individual mice expressed as fmol of immunoprecipitated ^125^I-αBuTx binding sites per gram of muscle is shown, with horizontal and vertical bars representing the mean and SD, respectively. Statistical analysis in **(C, D)** was performed using one-way ANOVA, corrected for multiple comparisons, and assays carried out as described in the Methods section. ns, not significant.

The efficacy of povetacicept was first evaluated in an EAMG experiment designed to assess the dose regimen and timing of povetacicept administration one week before or after the second AChR/IFA boost (chronic EAMG phase) (**Figure 1A**). Povetacicept treatment significantly ameliorated disease as compared to PBS-EAMG mice (open circles), either when dosing began at early signs of disease (Week 7; black squares) or when more severe EAMG disease signs were present (Week 9; grey squares). At the end of the experiment (day 88), the mean clinical EAMG score for the povetacicept Week 7 and Week 9 groups were significantly lower than the PBS-EAMG group (p=0.0447 and 0.0123, respectively) (**Figure 1A**). A significant reduction in serum antibodies specific for mouse AChR was observed in the povetacicept Week 9 group (mean 117.8 pmoles/ml ± 28.4 SD) as compared to the PBS-EAMG group (194.4 pmoles/ml ± 68.28 SD; p = 0.0442) (data not shown).

Next, the first of the two comparative therapeutic experiments was performed, in which the activity of povetacicept was compared to that of WT TACI-Fc (telitacicept), an Fc control, or PBS-EAMG mice (**Figure 1B**). Both povetacicept (dark grey squares)- and telitacicept (light grey squares)-treated mice showed a significant amelioration in EAMG disease signs as compared to the Fc control (grey circles) and PBS-EAMG (white circles) groups. The mean EAMG clinical score at the end of the experiment (Week 13) for the povetacicept group was 0.67 (± 0.13 SEM, p=0.0004 vs. PBS-EAMG and p <0.0001 vs. Fc control groups), for the telitacicept group was 0.83 (± 0.13 SEM, p=0.0088 vs. PBS-EAMG control and p=0.0010 vs. Fc control groups), for the Fc control group was 2.17 (± 0.17 SEM), and for the PBS-EAMG group was 1.9 (± 0.19 SEM). No significant difference was observed between mean clinical scores of PBS-EAMG and Fc control groups of mice.

Clinical improvement in povetacicept-treated mice was associated with a significant reduction in terminal serum levels of anti-mouse AChR antibodies as compared to PBS-EAMG, telitacicept, and Fc control groups (p=0.0038, p<0.0001, and p=0.0340, respectively), whereas telitacicept treatment did not significantly affect the anti-AChR antibody levels compared to the PBS-EAMG or Fc control groups (**Figure 1C**). Furthermore, muscle AChR content was significantly higher in mice treated with povetacicept compared to the PBS-EAMG group (p=0.0236) (**Figure 1D**). Telitacicept-treated animals also had increased muscle AChR content compared to the PBS-EAMG group though it did not reach statistical significance (p=0.0903). The muscle AChR content in the Fc control group was not statistically different from that in the PBS-EAMG group of mice.

In summary, therapeutic treatment with povetacicept reduced clinical manifestation of disease in the EAMG mouse model, which was associated with a significantly lower level of anti-AChR serum antibodies and higher content of muscle AChR.

### Povetacicept treatment decreases total serum immunoglobulins

**Figure 2:**
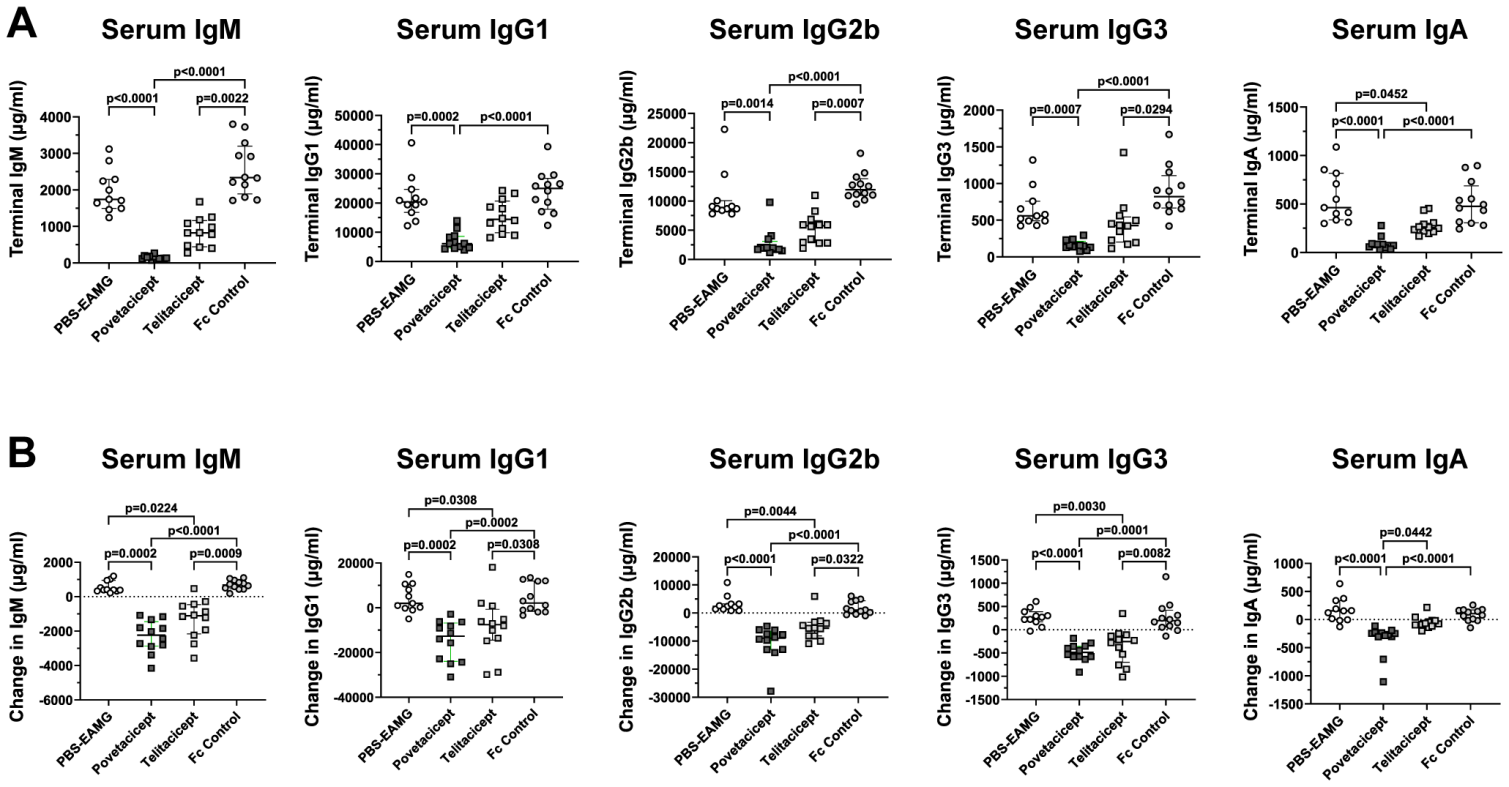
Povetacicept treatment reduces concentrations of total serum immunoglobulins. Total IgM, IgG1, IgG2b, IgG3, and IgA concentrations in serum samples collected just prior to the start of dosing (Week 9; “baseline”) and at termination (Week 13) from PBS-EAMG, povetacicept, telitacicept, or Fc control mice were measured using a magnetic bead multiplex kit as described in the Methods. Data from individual mice for terminal concentrations **(A)** and the difference in concentrations (terminal minus baseline) **(B)** are plotted, with horizontal and vertical bars representing the median and interquartile range, respectively. Statistical analysis was performed using the Kruskal-Wallis test with Dunn’s multiple comparisons test.

To further evaluate the activity of povetacicept in the EAMG model, its effect on total serum immunoglobulins of various subclasses was evaluated. As expected, there were no significant differences among the treatment groups for any of the immunoglobulin subclasses in serum samples collected at randomization, prior to the start of treatment (data not shown). On the other hand, analysis of serum samples collected at the end of the experiment (**Figure 2A**) and as a difference between values obtained at termination (Week 13) and just prior to the start of treatment (Week 9) (**Figure 2B**) demonstrated that povetacicept treatment resulted in significantly lower concentrations of all subclasses measured (e.g., IgM, IgG1, IgG2b, IgG3, and IgA) compared to PBS-EAMG and Fc control groups (**Figure 2**). The povetacicept group tended to have lower concentrations of all immunoglobulin subclasses than the telitacicept group, but the differences were not statistically significant except for the data set showing the difference in serum IgA from Week 13 to Week 9 (p=0.0442) (**Figure 2B**).

### Higher serum exposure and lower anti-drug antibodies with povetacicept treatment

**Figure 3:**
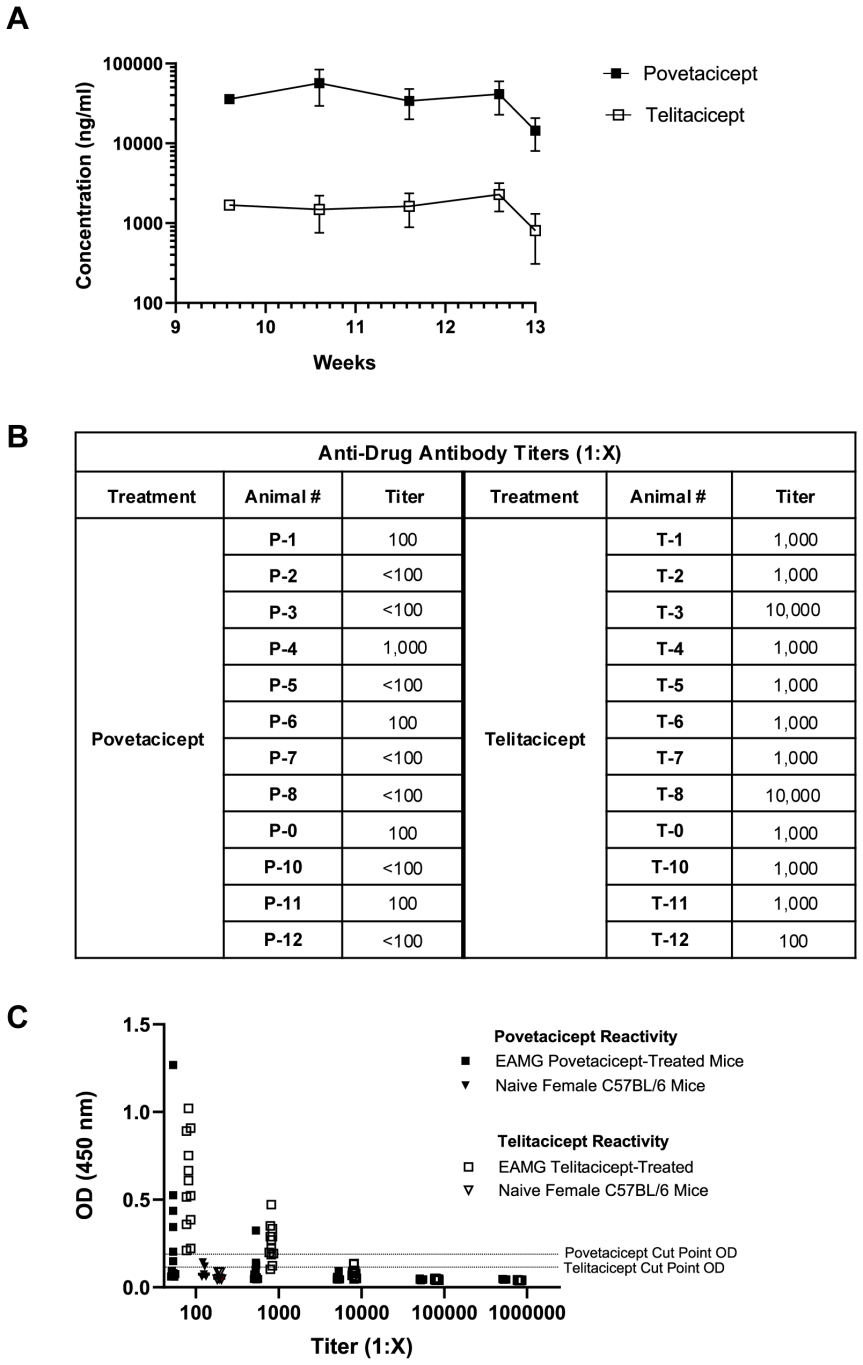
Serum exposure and anti-drug antibody levels. **(A)** Concentrations (ng/ml) of povetacicept and telitacicept in serum collected over time, just prior to administration of test articles at Week 9.6, 10.6, and 11.6, and at Week 12.6 and 13 (termination), measured using an ELISA as described in the Methods. Data are shown as the mean ± SD. **(B)** Anti-drug antibody (ADA) titers in individual mice from terminal serum, analyzed as described in the Methods. Titers <1:100 were considered undetectable. **(C)** Optical density (OD) values at 450 nm in the ADA ELISA for terminal serum samples from mice treated with povetacicept or telitacicept across all dilutions, or from naïve female C57BL/6 mice (n = 6) diluted 1:100 to obtain the cut-point OD. Data from individual mice are shown.

Serum concentrations of povetacicept and telitacicept collected during the treatment period indicated that, although there was little to no accumulation with the repeat dosing regimens, levels of povetacicept were at least 18-fold higher than those of telitacicept at all timepoints (**Figure 3A**). The mean concentrations of povetacicept and telitacicept were lower at termination than at other time points due to the longer post-dose period for the terminal bleed compared to other bleeds (168 hours vs. 96 hours). Terminal serum samples from povetacicept- and telitacicept-treated mice were also evaluated for ADA titers. Mice treated with povetacicept had lower titers than those treated with telitacicept (**Figure 3B**). OD values for individual mice across all dilutions are shown in **Figure 3C**.

### Secondary lymphoid organs are affected by povetacicept treatment

**Figure 4:**
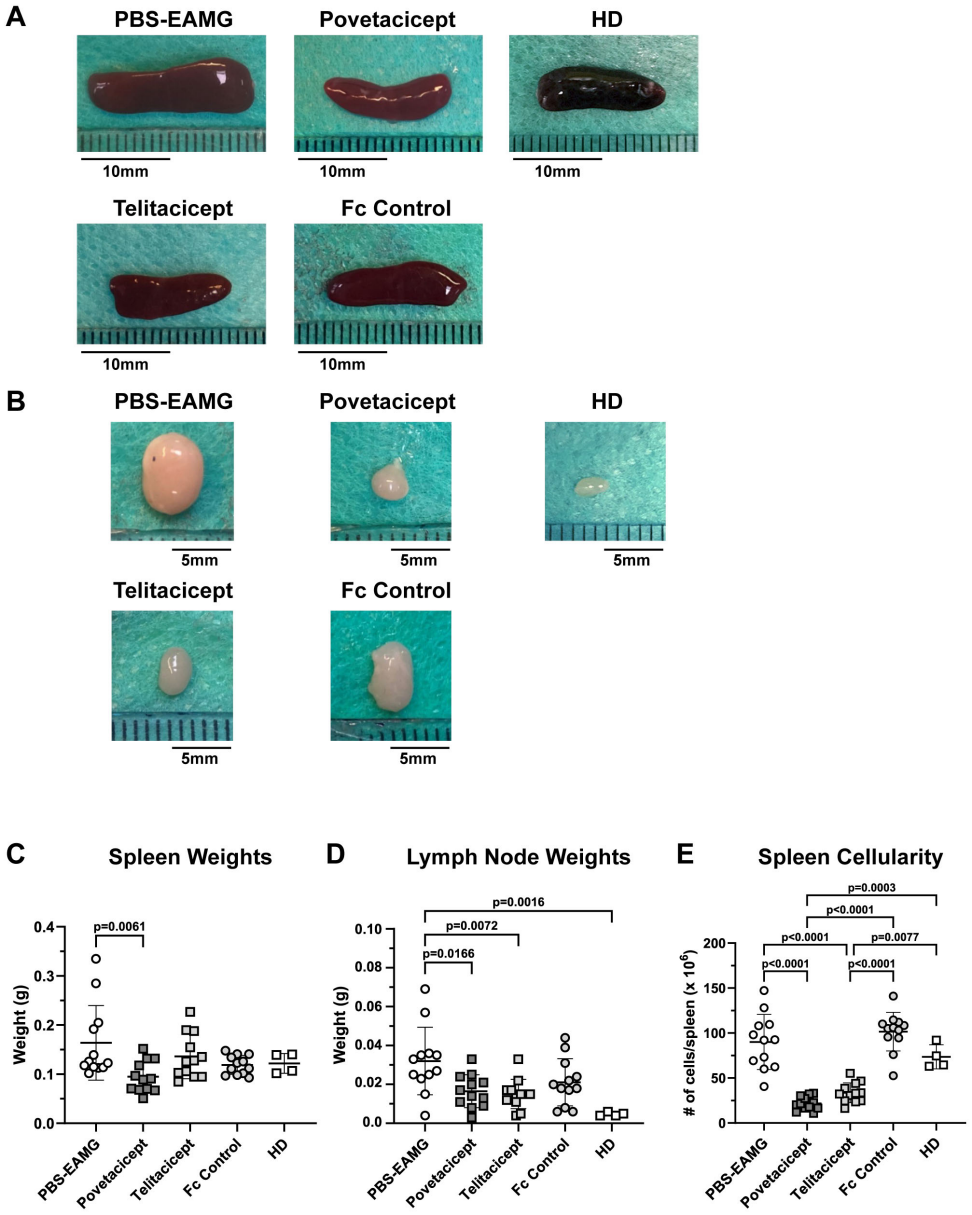
Secondary lymphoid organ weights are reduced in povetacicept-treated EAMG mice. Representative images of spleens **(A)** and draining (inguinal) lymph nodes **(B)** collected at termination (Week 13) are shown. Length of representative spleens are: PBS-EAMG controls = 19 mm, povetacicept = 15 mm, telitacicept = 14 mm, Fc control = 16.5 mm, healthy donor (HD) = 14.5 mm. Mean weights (expressed as grams) of spleens and LNs are presented in graphs **(C, D)**, respectively, for each treatment group (n=12 mice each) and for HD mice (n=4). **(E)** Total cellularity of spleens, processed for flow cytometry as described in the Methods. Individual mice are plotted, with horizontal and vertical bars as the mean and SD, respectively. Statistical analysis was performed using one-way ANOVA, corrected for multiple comparisons.

Immunization of animals with AChR in Freund’s adjuvant induces an increase in the size of secondary lymphoid organs, namely spleens and LNs draining the immunization sites (drLNs). Hence, representative images of spleens and drLNs harvested in the first comparator experiment are shown in **Figures 4A, B**, respectively, along with their weights (**Figures 4C, D**). Spleens and drLNs collected from the PBS-EAMG mice were grossly enlarged compared to those from healthy donor (HD) mice; spleen and drLN dimensions from povetacicept mice were significantly lower than those from the PBS-EAMG mice (**Figures 4A, C**). In addition, the mean spleen weight of the PBS-EAMG group was significantly greater compared to the povetacicept-treated mice (**Figure 4B**). Similarly, drLNs (**Figure 4D**) from PBS-EAMG mice were significantly heavier compared to those from the povetacicept group (**Figure 4D**). Mice treated with povetacicept or telitacicept had significantly reduced total spleen cellularity compared to the PBS-EAMG and the Fc control groups (**Figure 4E**).

### Effect of povetacicept on draining LN B cell follicles

**Figure 5:**
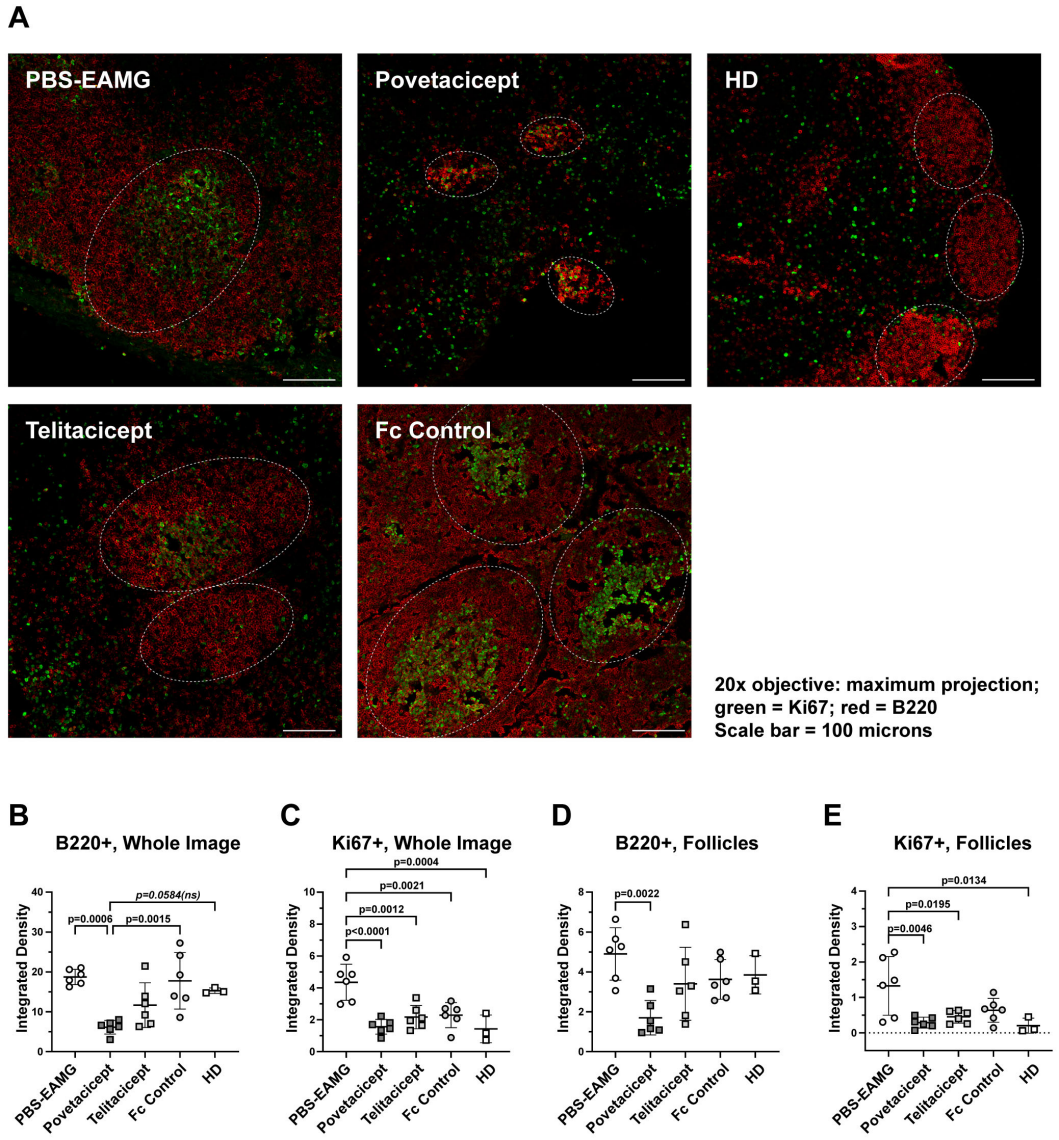
Povetacicept treatment results in significantly lower B cells and proliferating (Ki67^+^) cells within draining LNs (drLN). **(A)** Representative images of immunofluorescence on drLN, with maximum intensity projection of z-stack images (objective 20X). The drLNs were stained and imaged for Ki67+ cells (green) and B220+ cells (red) as described in Methods. The regions of interest (ROI) are delimited by white dotted lines and shown graphically in **(B-E)**. HD = healthy donor [mice]. Integrated densities of B220^+^ and Ki67^+^ cells in whole immunofluorescence microscopy images (**B, C**, respectively) and in the region of interest (**D, E**, respectively) were determined as described in the Methods section. Individual mice are plotted, with horizontal and vertical bars as the mean and SD, respectively. Statistical analysis was performed using one-way ANOVA, corrected for multiple comparisons. ns, not significant.

Draining LNs from a subset of experimental mice (n = 6/treatment group, chosen at random) were subjected to immunofluorescence analysis to evaluate B cell distribution and follicles via staining of B220^+^ cells (red fluorescence) and for Ki67, a marker of proliferating cells (green fluorescence). Statistical analysis of EAMG scores for each treatment group for all mice (**Supplementary Figure 2A**) compared to EAMG scores for the subset selected for immunofluorescence analysis (**Supplementary Figure 2B**) indicated that the random sampling of animals for immunofluorescence analysis did not induce any bias as there were no significant differences for any of the groups between the 2 data sets (p=0.95 to >0.99) (**Supplementary Figure 2**). Representative images are shown in **Figure 5A**. Analysis of whole microscopy images, performed with Fiji software, revealed a significant reduction in B220^+^ integrated density in the povetacicept group compared to the PBS-EAMG and Fc control groups (p=0.0006 and p=0.0015, respectively) and a non-significant reduction compared to the HD group of mice (p=0.0584) (**Figure 5B**). The B220^+^ integrated density tended to be lower in the telitacicept group of mice compared to the PBS-EAMG and Fc control groups but did not reach statistical significance (p=0.0809 and p=0.1641, respectively) (**Figure 5B**). Povetacicept and telitacicept treatments also decreased overall cell proliferation (measured via analysis of Ki67^+^ cells) compared to the PBS-EAMG group (p<0.0001 and p=0.0012, respectively) (**Figure 5C**). Immunofluorescence analysis was then focused on B cell follicles, identified as ROIs of B220^+^ cells (representative ROIs are shown in **Figure 5A**), revealing a significant reduction in B220^+^ integrated density in povetacicept-treated mice compared to PBS-EAMG (p=0.0022) and a non-significant reduction compared to the Fc control and HD groups (p=0.1033 and p=0.1589, respectively) (**Figure 5D**), indicative of smaller B cell follicles following povetacicept treatment. A higher integrated density mean value of Ki67^+^ cells (within the identified ROIs) was observed in PBS-EAMG mice compared to povetacicept (p=0.0046), telitacicept (p=0.0195), Fc control (p=0.0891), and HD mice (p=0.0134) (**Figure 5E**). Together, these data indicate that povetacicept treatment effectively reduced B220^+^ cell population and Ki67^+^ proliferating cells in the drLN B cell follicles.

### Povetacicept administration modifies splenic T and B cell populations

**Figure 6:**
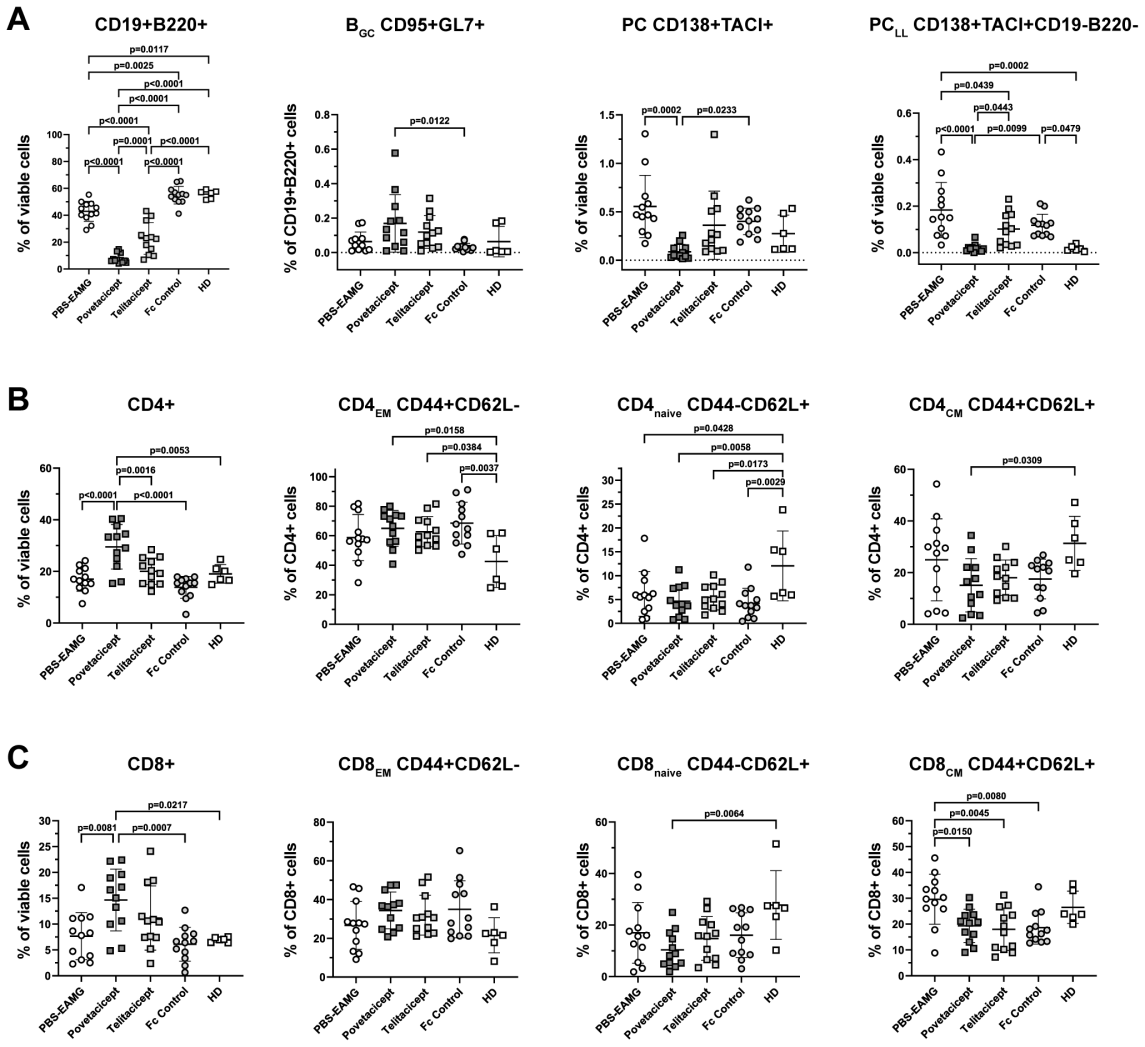
Povetacicept treatment modifies B and T cell subset compositions in spleens. **(A)** Flow cytometry analysis of CD19^+^B220^+^ (% of viable cells), B_GC_ CD95^+^GL7^+^ (% of CD19^+^B220^+^ cells), PC CD138^+^TACI^+^ (% of viable cells), PC_LL_ CD138^+^TACI^+^CD19-B220- (% of viable cells). **(B)** CD4+ T cell and **(C)** CD8+ T cell compartments were analyzed to detect changes in CD4_EM_ (CD44^+^CD62L^-^), CD4_naive_ (CD44^-^CD62L^+^), CD4_CM_ (CD44^+^CD62L^+^) and CD8_EM_ (CD44^+^CD62L^-^), CD8_naive_ (CD44^-^CD62L^+^), CD8_CM_ (CD44^+^CD62L^+^). n=12 spleens/treatment group collected at termination (Week 13); healthy donor (HD) = 6 spleens. Individual mice are plotted, with horizontal and vertical bars as the mean and SD, respectively. Statistical analysis was performed using one-way ANOVA, corrected for multiple comparisons.

To further evaluate the effects of povetacicept and potential mechanisms of its efficacy, immune cell phenotyping of splenocytes was conducted by flow cytometry to assess differences in cell subset frequencies in EAMG mice in the first therapeutic experiment (**Figure 6**). Since cells needed to be frozen for flow cytometry analysis at a later timepoint due to logistical constraints (i.e., since terminations in each experiment were dictated by the variable time for mice to reach terminal time points), it was not possible to obtain an accurate count of total/subset cell numbers that would be representative of those in fresh spleens.

The analysis showed a significant reduction in the percentage of CD19^+^B220^+^ B cells in the povetacicept treatment group compared to the PBS-EAMG, telitacicept, and Fc control groups of mice (p ≤ 0.0001 for all comparisons) (**Figure 6A**). Telitacicept treatment also resulted in a significantly lower percentage of B cells compared to the PBS-EAMG group (p<0.0001), though to a lesser extent than with povetacicept treatment. The percentage of B_GC_ cells (CD95^+^GL7^+^ gated on B cells) was increased in the povetacicept group compared to Fc control treatment (p=0.0122). Mice treated with povetacicept showed a statistically significant lower percentage of PC (CD138^+^TACI^+^ cells from the live cell gate) compared to the PBS-EAMG (p=0.0002) and Fc control (p=0.0233) groups. Povetacicept treatment also resulted in a lower percentage of the PC_LL_ population (CD19^-^B220^-^ cells within the CD138^+^TACI^+^ PC gate) compared to PBS-EAMG (p<0.0001) and Fc control mice (p=0.0099). Telitacicept treatment induced a decrease in PC_LL_ compared to the PBS-EAMG (p=0.0439), though to a lesser extent than with povetacicept treatment (**Figure 6A**).

The effect of povetacicept treatment was also evaluated on both CD4^+^ and CD8^+^ T cell subsets. The frequency of CD4^+^ cells (as a percentage of viable cells) was significantly higher in povetacicept-treated mice compared to PBS-EAMG (p<0.0001), telitacicept (p=0.0016), and Fc control (p<0.0001) groups. Although some significant differences were observed compared to the HD group of mice, there were no significant differences between treatment groups for CD4_EM_, CD4_naïve_, or CD4_CM_ subsets (**Figure 6B**), nor for follicular and regulatory CD4 T cell subsets (data not shown). Similarly, the frequency of CD8^+^ T cells, as a percentage of viable cells, was higher in the povetacicept group compared to PBS-EAMG (p=0.0081) and Fc control (p=0.0007) groups; in povetacicept-, telitacicept-, and Fc control-treated mice, a reduced percentage of CD8_CM_ T cells was observed compared to the PBS-EAMG group (p=0.0150, p=0.0045, and p=0.0080, respectively)(**Figure 6C**). Although absolute cell numbers were not quantified in these studies, the changes in T cell frequencies observed in the povetacicept-treated mice likely reflect proportional increases due to the reduced B cell frequencies, as has been observed in previous preclinical studies with povetacicept (15).

### Comparison of povetacicept treatment with the neonatal Fc receptor blocker efgartigimod and anti-CD20 antibody

**Figure 7:**
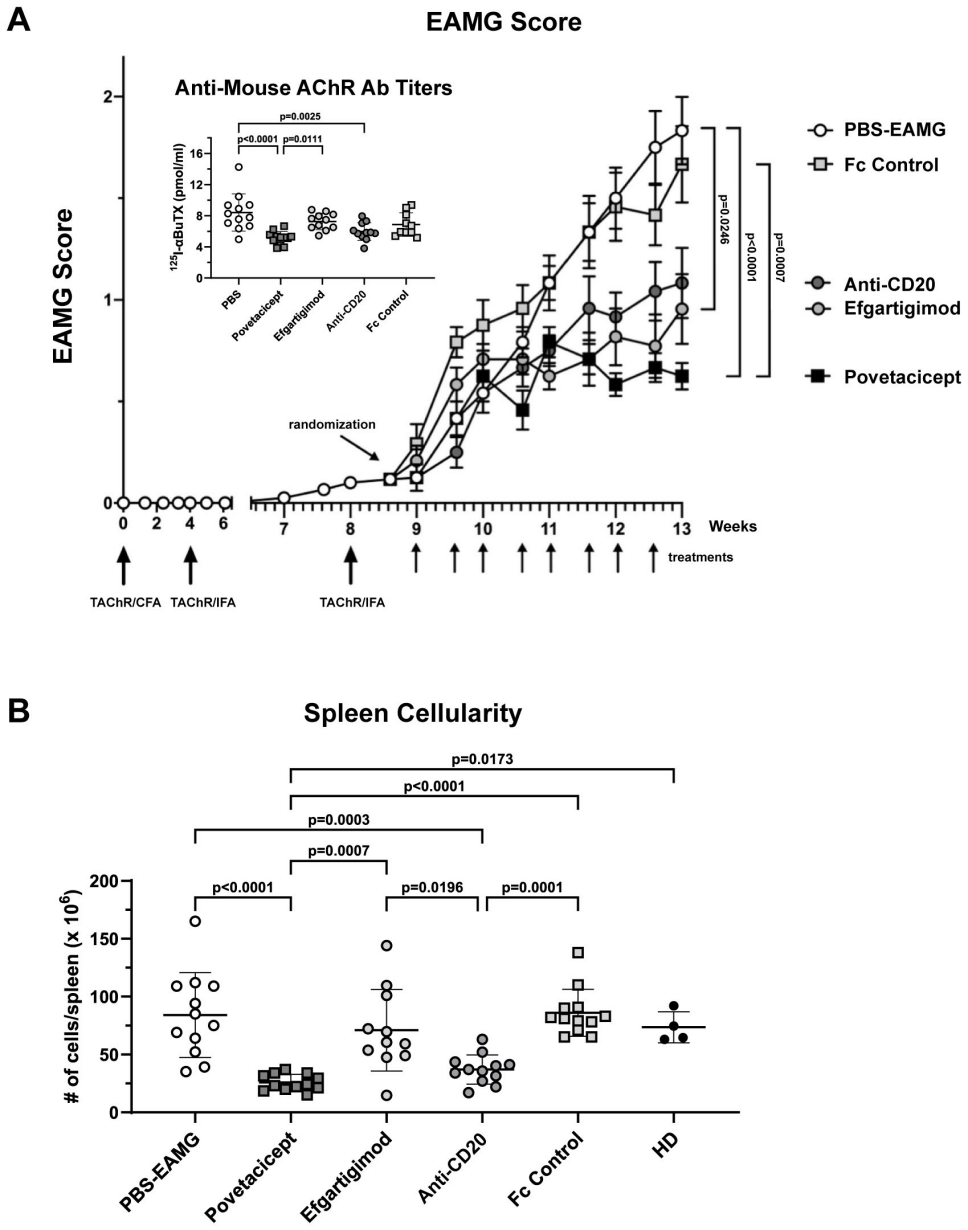
Povetacicept efficacy in ameliorating clinical signs of EAMG. **(A)** EAMG mice received povetacicept (black squares, n=12 mice, twice weekly for 8 doses total), efgartigimod (light grey circles, n=12 mice, 8 doses total), anti-CD20 (dark grey circles, n=12, once weekly for 4 doses total), Fc control protein (light grey squares, n=12 mice, 8 doses total), or PBS for the PBS-EAMG group (white circles, n=12 mice, 8 doses). TAChR immunization and boosts are indicated with the larger upward arrows and i.p. treatments with smaller upward arrows along the x-axis. Statistical significance of clinical scores was calculated using the Kruskal-Wallis test with Dunn’s multiple comparisons test; data are presented as mean EAMG scores (± SEM). Insert: Anti-mouse AChR antibody titers in individual mice (pmoles of immunoprecipitated ^125^I-αBuTx binding sites per ml of serum). **(B)** Total cellularity of spleens collected at termination (Week 13) and processed for flow cytometry as described in the Methods. Anti-mouse AChR Ab and spleen cellularity data are presented as scattered dot plot graphs of individual mice, with horizontal and vertical bars representing the mean and SD, respectively. Statistical analysis was performed using one-way ANOVA, corrected for multiple comparisons.

To further assess the efficacy of povetacicept in ameliorating EAMG, a second comparative therapeutic experiment was conducted with additional relevant therapeutic molecules (**Figure 7**). EAMG mice were treated twice weekly with povetacicept, Fc control, or PBS compared with efgartigimod (twice weekly) and anti-CD20 (once weekly), beginning one week after the second boost (Week 9). As observed in the first comparator experiment, mice that received povetacicept exhibited significantly milder EAMG disease compared to PBS-EAMG mice (p<0.0001) and Fc control-treated mice (p=0.0007) at the end of the experiment (Week 13). Efgartigimod treatment significantly reduced disease scores compared to the PBS-EAMG group (p=0.0246) but not compared to the Fc control treatment group (p=0.1076), whereas anti-CD20 treatment did not significantly ameliorate clinical signs compared to either PBS or Fc control (p=0.1077 and p=0.3927, respectively). No additional differences were observed among treatment groups.

Povetacicept treatment also resulted in significantly lower serum levels of anti-mouse AChR antibodies compared to the PBS-EAMG (p<0.0001) and efgartigimod-treated mice (p=0.0111); anti-AChR titers were also significantly decreased by anti-CD20 (p=0.0025) treatment compared to the PBS-EAMG group of mice but not with efgartigimod treatment (**Figure 7** insert).

**Figure 8:**
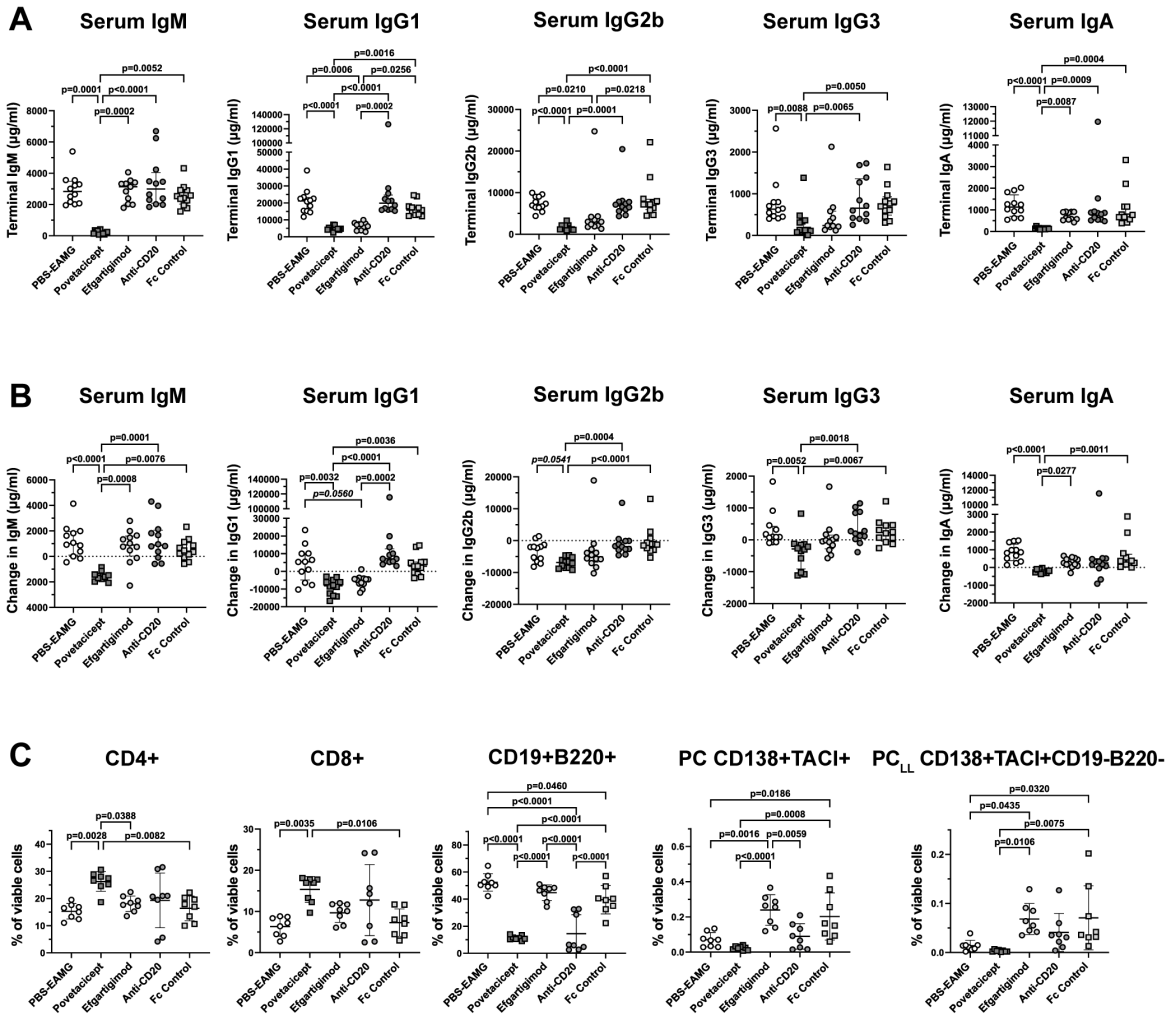
Povetacicept reduces serum concentrations of total immunoglobulins and impacts splenic T and B cell frequencies. **(A)** Total IgM, IgG1, IgG2b, IgG3, and IgA concentrations were measured in serum samples collected just prior to the start of dosing (Week 9; “baseline”) and at termination (Week 13) from EAMG mice treated with PBS (EAMG controls), povetacicept, efgartigimod, anti-CD20, or Fc control. Concentrations from individual mice for terminal concentrations **(A)** and the difference in concentrations (terminal minus baseline) **(B)** are plotted, with horizontal and vertical bars representing the median and interquartile range, respectively. Statistical analysis was performed using the Kruskal-Wallis test with Dunn’s multiple comparisons test; p values for significant comparisons (p<0.05) are shown. **(C)** Flow cytometry analysis of T cell and B cell subsets in splenocytes (n=8 mice/group) collected at termination (Week 13). Data for individual mice are plotted, with horizontal and vertical bars representing the mean and SD, respectively. Statistical analysis was performed using one-way ANOVA, corrected for multiple comparisons.

Significantly lower concentrations of total IgM, IgG1, IgG2b, IgG3, and IgA in terminal serum samples (**Figure 8A**) and in concentrations calculated as the difference between Week 13 (termination) and Week 9 (start of treatment) (**Figure 8B**) were observed in povetacicept-treated mice compared to the PBS-EAMG, anti-CD20, and Fc control groups, as well as significantly lower concentrations of serum IgM and IgA compared to the efgartigimod treatment group (**Figures 8A, B**).

Flow cytometry analysis of splenocytes in a subset of mice (n=8 mice/group) confirmed results obtained in the first comparator experiment (**Figure 8C**). Mice treated with povetacicept had a higher percentage of CD4^+^ and CD8^+^ T cells compared to PBS-EAMG (p=0.0028 and p=0.0035 for CD4^+^ and CD8^+^ T cells, respectively) and Fc control (p=0.0082 and 0.0106 for CD4+ and CD8+ T cells, respectively) groups. Povetacicept treatment induced a significant reduction in CD19^+^B220^+^ B cells compared to the PBS-EAMG (p<0.0001), efgartigimod (p<0.0001), and Fc control (p<0.0001) groups (**Figure 8C**). Mice treated with anti-CD20 also had a significantly lower percentage of CD19^+^B220^+^ B cells compared to PBS-EAMG mice (p<0.0001), Fc control (p<0.0001), and efgartigimod (p<0.0001) groups but to a lesser extent and with greater variability than observed with povetacicept treatment. Povetacicept treatment resulted in significant reductions in the frequencies of PC compared to Fc control (p=0.0008) and efgartigimod (p<0.0001) groups, and also significantly reduced PC_LL_ frequencies compared to efgartigimod (p=0.0106) and Fc control (p=0.0075) groups. A greater variability in PC and PC_LL_ frequencies was observed in efgartigimod and Fc control groups compared to the PBS-EAMG group. No differences were observed in B_GC_ cell percentages among groups (data not shown).

## Discussion

Povetacicept has been recently investigated for its ability to suppress B cell proliferation, differentiation, and Ig secretion both in *in vitro* B cell cultures and in a mouse SLE model, demonstrating a significant reduction of serum Ig levels and antibody-secreting cells (15). As B cells (including antibody secreting cells) and immunoglobulins have been shown to play multiple important roles in MG pathogenesis (6, 29), we have evaluated the efficacy of povetacicept in modulating clinical manifestations in the mouse EAMG model in the studies described herein, by comparing povetacicept treatment to (i) telitacicept, (ii) anti-CD20 (B cell depleting) mAb, (iii) efgartigimod (FcRn blocker), (iv) Fc control, and (v) PBS (vehicle), administered in the chronic EAMG phase (after the second AChR/IFA boost). The effectorless Fc control protein used in these studies was matched to povetacicept’s Fc and was intended to enable comparison of effects of an inert human protein to those induced by the active TACI-Fc BAFF/APRIL inhibitors povetacicept and telitacicept. In these and previous studies, including *in vitro* studies with human cells (15), we have observed some inter-experiment variation in the Fc control effects on the lymphoid compartment. Herein, the average %CD19^+^B220^+^ B cells in the Fc control group in Comparator Experiment #1 was slightly higher than in the PBS group (**Figure 6A**; p=0.0025) whereas the reverse was true in Comparator Experiment #2 (**Figure 8C**; p=0.046). However, these modest differences fall within the variation expected for these *in vivo* models based on our prior experience (15) and do not impact the interpretation of the overall results.

Our data demonstrated povetacicept efficacy in modulating EAMG manifestation (**Figures 1A, B**, **7**), along with a significant reduction of pathogenic anti-mouse AChR autoAb titers and increased muscle AChR contents compared to PBS-EAMG and Fc control groups (**Figures 1C, D**, **7**), and to the efgartigimod treatment group (**Figure 7**). As AChR immunization in Freund’s adjuvant induces a robust immune system activation with morphological alterations in drLNs and spleen, we performed cellular analysis on cryopreserved organs collected from experimental mice. The analysis showed that drLNs and spleens from povetacicept-treated mice were significantly smaller compared to the PBS-EAMG group (**Figure 4D**), in parallel with a significantly lower yield of splenocytes from single cell suspensions (**Figure 4E**), confirming the important role of BAFF and APRIL in mediating B cell activation and expansion in these organs following immune sensitization with AChR.

Immunofluorescence analysis was conducted on drLNs to identify mature B cells and follicles. As reported by Pikor and collaborators (30), the B220 surface marker identifies mature B cells and distinguishes follicle areas with activated and proliferating B cells, subsequently differentiating into central memory B cells or PC (31). Our analysis on drLNs revealed that blocking BAFF and APRIL signalling results in a decreased B220^+^ integrated density compared to PBS and Fc control treatments, and more specifically in B cell follicles, in parallel with lower Ki67^+^ signal compared to the PBS-treated group (**Figure 5**); these data further strengthen our hypothesis that the inhibition of BAFF and APRIL dampens B cell activation. Since Ki67 protein is a marker for cellular activation and proliferation, it is also expressed by other cell types involved in the sensitization processes to AChR, and we cannot exclude that BAFF and APRIL may influence the status of follicular helper CD4 T cells, dendritic cells, and other immune cell subsets (32).

BAFF and APRIL are cytokines that promote differentiation, survival, and function of B cells at different stages of B cell maturation (33). Indeed, our observations in spleens (**Figure 6**) revealed that not only are CD19^+^B220^+^ cell percentages significantly reduced with povetacicept treatment but also PC and PC_LL_ subsets (**Figure 6A**), similar to previous observations with povetacicept (15). The observed increase in the frequency of CD4^+^ (**Figure 6A**) and CD8^+^ T cells as a percentage of viable cells (**Figure 6B**) in spleens of povetacicept-treated EAMG mice, compared to the PBS-EAMG and other treatment groups, is likely due to the proportionally decreased percentage of B220^+^CD19^+^ B cells.

Both povetacicept and telitacicept were able to modulate EAMG manifestations, but telitacicept, at the dose evaluated (equimolar to povetacicept) was less effective at reducing total serum immunoglobulins (**Figure 2**) and anti-AChR autoAbs, and at maintaining muscle AChR content (**Figures 1C, D**). Of note, EAMG mice received equimolar and saturating amounts of povetacicept or telitacicept, but the resulting serum levels of povetacicept were at least 18-fold higher than those of telitacicept, which is consistent with our previous observations of povetacicept vs. telitacicept exposure in other mouse disease models (**Figure 3A** and K.E.L., unpublished results). Although the titers and incidence of ADA were higher in the telitacicept-treated EAMG mice at termination (5/12 povetacicept-treated mice were ADA+ with titers ranging from 1:100–1000 vs. 9/12 ADA+ mice in the telitacicept group with titers ranging from 1:100-10,000) (**Figure 3B**), those findings do not fully explain the differences in exposure observed relatively soon after dosing (4 days after the first dose). Similar differences in exposure between povetacicept and telitacicept have also been observed in cynomolgus monkeys (15) and may reflect the increased target affinity and consequently slower clearance of povetacicept, which may enable longer dosing intervals in the clinic. We have consistently observed enhanced efficacy of povetacicept over telitacicept in multiple mouse disease models (15), likely driven by a much slower off-rate than that observed for telitacicept and other WT TACI-Fc molecules (15). This slow off-rate enables povetacicept to engage its targets longer than WT TACI-Fc, leading to greater neutralization of BAFF/APRIL signalling to mediate deeper and more prolonged pharmacodynamic effects, including more thorough reductions in Ig class switching, antibody-secreting cell survival, and immunoglobulin and autoantibody levels. Notably, BAFF/APRIL inhibition can impact B cell functions beyond Ig production, including reductions in antigen presentation to autoreactive T cells and cytokine production (34).

It is of interest to assess these findings in the context of the clinical treatment of MG patients and reflect on the translatability of the EAMG model to clinical MG. Treatment modalities currently used to reduce the levels of pathogenic IgG autoAbs in MG include corticosteroids, immunosuppressants, B cell-targeting drugs, IVIG infusions, plasma exchange, and immunoadsorption (35, 36). However, these treatment options have different degrees of deficiencies in terms of safety, efficacy, and accessibility. Conventional immunosuppressive drugs may provide some efficacy, but their use can be limited by adverse reactions (37). B cell targeting therapies like rituximab (anti-CD20 mAb), belimumab (anti-BAFF mAb), and WT TACI-Fc fusion proteins (atacicept and telitacicept) have demonstrated promising therapeutic potential in various clinical studies, particularly for lupus, given its strong association with pathogenic B cells (38–45). However, to date, no B cell targeted therapies have been approved for the treatment of MG. Rituximab has been used off-label in MG and is used in anti-MuSK patients who are refractory to conventional treatments (46). In addition, the international consensus guidelines, most recently revised in 2021, recommend rituximab as an early treatment for these patients (37).

Targeting BAFF and/or APRIL presents a relatively new approach to depleting autoAb-producing plasma cells, most of which are exquisitely dependent upon both cytokines (47). Long-lived plasma cells stably maintain serum antibody levels, whereas memory B cells are responsible for recall responses upon antigen re-exposure. CD20 has an important role in the growth and differentiation of B cells into plasma cells, and rituximab can efficiently deplete CD20-positive B cells in MG patients; however, rituximab is ineffective in reducing pathogenic AChR-Ab levels (48). Long-lived plasma cells are the major producers of anti-AChR autoAb and lack CD20, hence rituximab targets only short-lived plasma cells still expressing CD20 and CD20^+^ IL-10-producing regulatory B cells, and therefore reduction of autoAb is generally short term and insufficient, resulting in only transient clinical improvement (6, 49). BAFF/APRIL inhibitors may therefore enable longer term reductions in autoAb and associated prolonged clinical responses.

The importance of BAFF itself in MG has been evaluated preclinically and clinically, and higher BAFF levels in MG patients have been reported (9, 50, 51). Although serum BAFF levels did not correlate with MG disease severity, a significant correlation was observed between serum BAFF levels and anti-AChR antibody titers (51). BAFF has also been implicated in the formation and maintenance of ectopic germinal centers in the thymus, in concert with the chemokine CXCL13, possibly revealing an important role for BAFF in pathogenic B cell homeostasis in MG (52, 53). Although a belimumab surrogate (i.e., anti-mouse BAFF mAb) has not yet been evaluated in the experimental MG models, treatment of EAMG mice with low doses of a BAFF-R-specific mAb-siRNA conjugate, designed to mediate depletion of BAFF-R^+^ B cells, was associated with EAMG amelioration without affecting autoAb levels (54). Of note, a Phase 2 study evaluating the efficacy and safety of belimumab in generalized MG patients was conducted but did not meet the primary endpoint, suggesting that BAFF-only inhibition is insufficient to drive clinical responses (55).

Indeed, inhibition of either BAFF or APRIL alone in various other preclinical models mediates relatively modest effects, whereas their co-neutralization dramatically reduces B cell differentiation and function, including reductions in antibody/autoAb production (15, 56–58). Since serum levels of APRIL have also been found to be elevated in MG patients relative to healthy controls (10), APRIL and BAFF signalling may play overlapping but non-redundant roles in MG. Local expression of both BAFF and APRIL in the thymus of patients with MG may create an environment that facilitates B cell survival, thereby sustaining production of anti-AChR antibodies (52). Hence, dual antagonism of both BAFF and APRIL may confer greater efficacy and improved clinical outcomes in the treatment of MG compared with available therapeutics. To that point, case reports have been described of telitacicept efficacy in AChR^+^ MG patients (59–61). Given that povetacicept has demonstrated improved BAFF/APRIL inhibition with superior pharmacokinetic and pharmacodynamic effects compared to telitacicept and other WT TACI-Fc proteins in preclinical (15) and clinical settings (18), including in the EAMG studies described here, the enhanced TACI-Fc fusion protein povetacicept may achieve superior outcomes in B cell-mediated autoimmune diseases like MG.

The limitations of these studies include the inherent caveats associated with any animal model for human disease. The active induction EAMG model studied here is considered the one best suited for preclinical evaluation of therapeutics for MG that affect B cell and autoantibody development pathways, when administered after the onset of symptoms. Although the model cannot fully reproduce human MG pathology, the active EAMG model is a well-regarded and useful tool for the preclinical evaluation of mechanisms-of-action and comparability of therapeutics. Muscle AChR content was measured at termination, which is a well-accepted, objective, and appropriate measure of EAMG disease (19, 20). It may have been informative to also measure the change in muscle AChR content as an effect of treatment, though it was not possible to accurately measure this outcome longitudinally due to the methodology to assess muscle AChR content (i.e., from carcasses) which would have required the use of a much greater number of animals. We did include EAMG clinical scoring of the mice over the entire course of the study, for which the scorer was blinded to the treatments. This is also a well-accepted and validated method of EAMG disease assessment, which includes a semi-quantitative evaluation of muscle strength and disease progression (19). Other functional tests exist for EAMG models (e.g., grip strength, rotarod performance, wire hang tests) though, in our experience, the tests used in the current studies provide accurate data with the least amount of bias. The variability in the anti-mouse AChR titers and plasma cell frequencies amongst the 3 experiments reflects the normal variation observed in this active EAMG model, likely due to several factors including different lots or administration of even slightly different amounts of the TAChR immunogen (62–64). Despite the experimental variations in anti-AChR titers, there was a significant signal above the baseline (i.e., naïve mice had no detectable anti-AChR antibodies), and we confirmed that the mice developed significant EAMG disease using neostigmine (an anti-cholinesterase inhibitor), which results in temporary reversal of clinical signs (20). In fact, autoAb variability is also observed clinically, where antibody levels vary dramatically and are not always associated with disease severity (reviewed in (65)), but their reduction is associated with effective treatment (66). It is in fact notable that povetacicept was able to significantly reduce anti-AChR antibodies across a range of levels.

Regarding the flow cytometry methodology and results, due to logistical constraints associated with study timelines, splenocytes needed to be frozen in order to analyze all study samples together on the cytometer and avoid day-to-day variability. The required freeze-thaw of splenocytes precluded our ability to calculate total numbers of immune cell subsets that would be fully representative of the cellular composition of fresh spleens, though previously published preclinical studies with povetacicept that included its effects on total cell numbers (15) help support the conclusions made with the cell subset frequency data presented here.

We have defined long-lived plasma cells in our EAMG studies using a previously described immunophenotype (i.e., viable TACI^+^CD138^+^B220^–^CD19^–^ cells) (28), though we did not formally confirm the functionality of cells bearing this phenotype in our experiments or employ additional markers. Future studies could include such markers (i.e., Sca-1, CD98, ITGA4, CD94, etc.) and/or assays to evaluate PC functionality.

In addition, quantification of APRIL and BAFF in serum was not possible in the studies presented here due to protein stability and serum volume limitations, though alternative data were used to select the dosing regimen to ensure full target coverage by the TACI-Fc molecules. The dose regimen for povetacicept used in these studies (10 mg/kg twice a week) was selected based on a mouse dose response PK/PD study that indicated that this regimen saturated the expected pharmacodynamic effects, including reductions in Ig and B cells. Calculations of the human equivalent dose, adjusted for the ~5-7-fold reduced potency of povetacicept for mouse BAFF and APRIL *in vitro* (15), predict that the dosing regimen used in the EAMG mouse model is roughly equivalent to 90 mg every 4 weeks in humans, which is similar to the 80 mg every 4 weeks dose regimen being evaluated in the ongoing Phase 3 trial in IgA nephropathy (NCT06564142). To enable a direct comparison of efficacy between the TACI-Fc test articles, we used a molar matched dose of telitacicept (~12 mg/kg twice a week) to ensure delivery of the same number of binding sites for telitacicept as for povetacicept. Along with evidence supporting povetacicept’s potent APRIL and BAFF inhibition as compared to WT TACI-Fc (i.e., telitacicept), we have published preclinical *in vitro* and *in vivo* data demonstrating greater potency of povetacicept compared to individual APRIL and BAFF inhibitors (15). It would be of interest to include individual BAFF and APRIL inhibitors in future preclinical studies of EAMG. Increased expression of BAFF and its receptors have been demonstrated in the thymus of patients with MG, with implications for altered cellular output that may contribute to disease (12, 52, 53, 67, 68). Although we did not evaluate the cellular composition of the thymus in the studies described herein due to logistical limitations, this could be an interesting endpoint to study in future EAMG experiments.

In conclusion, the classical EAMG model in rodents studied herein is considered relevant to investigate novel therapeutic modalities for MG (22, 69) and indeed, the mouse EAMG model has been previously used to evaluate the efficacy of several clinical candidates, including TNFR-Fc (70), teriflunomide (71), and anti-IL-23 (72). Our results in the EAMG mouse model demonstrate that povetacicept treatment modulates the immune response to AChR and counteracts the progression of the disease, with comparable or better efficacy than clinically relevant therapeutics. Further, these data suggest that povetacicept may represent an effective and innovative molecule to treat MG and support its evaluation in future clinical trials.

## Data Availability

The raw data supporting the conclusions of this article will be made available by the authors, without undue reservation.

## Glossary

AChR: acetylcholine receptor
ADA: anti-drug antibodies
APRIL: a proliferation-inducing ligand
autoAb: autoantibody(ies)
BAFF: B-cell activating factor
BAFF-R: B-cell activating factor receptor
BCMA: B-cell maturation antigen
B_GC_: germinal center B cells
BuTX: bungarotoxin
CD4_CM_: central memory CD4 T cells
CD4_EM_: effector memory CD4 T cells
CD4_naive_: naive CD4 T cells
CD8_CM_: central memory CD8 T cells
CD8_EM_: effector memory CD4 T cells
CD8_naive_: naive CD8^+^ T cells
CFA: complete Freund’s adjuvant
CXCR5: C-X-C chemokine receptor 5
EAMG: experimental autoimmune myasthenia gravis
EDTA: ethylenediaminetetraacetic acid
EGTA: ethylene glycol-bis(β-aminoethyl ether)-N,N,N′,N′-tetraacetic acid
FcR: Fc receptor
FcRn: neonatal Fc receptor
FMO: fluorescence-minus-one
GC: germinal center
IFA: incomplete Freund’s adjuvant
LN: lymph node(s)
LRP4: lipoprotein receptor related protein 4
MFI: mean fluorescence intensity
MG: myasthenia gravis
MuSK: muscle-specific kinase
NMJ: neuromuscular junction
OD: optical density
PC: plasma cells
PC_LL_: long-lived plasma cells
PMBC: peripheral blood mononuclear cell
PMSF: phenylmethylsulfonyl fluoride
ROI: region of interest
TAChR: Torpedo acetylcholine receptor
TACI: transmembrane activator and CAML interactor
TNF: tumor necrosis factor
WT: wild type
