# Supplementary material for: Povetacicept (ALPN-303; TACI vTD-Fc), an enhanced, potent dual inhibitor of BAFF and APRIL, ameliorates experimental autoimmune myasthenia gravis in C57BL/6N mice
Source: Front Immunol. 2025 Jun 6;16:1533093. doi: 10.3389/fimmu.2025.1533093 (PMC12179661; doi:10.3389/fimmu.2025.1533093)
Supplement: Supplementary file 1 [file Presentation1.pdf]

A Pilot Experiment

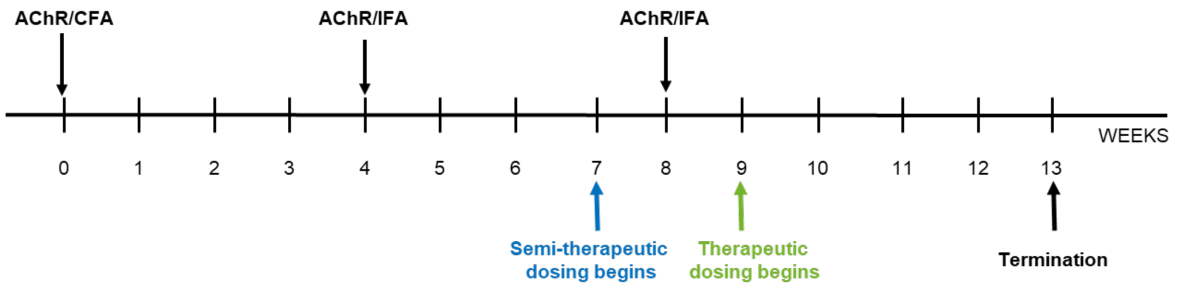

Treatment Groups (i.p. dosing):

1. PBS (n = 5); twice weekly from Week 7 to 11.6.
2. Povetacicept (~10 mg/kg; n = 5); twice weekly from Week 7 to 11.6 (“semi-therapeutic”).
3. Povetacicept (n = 5); twice weekly from Week 9 to 11.6 (“therapeutic”).

B Therapeutic Comparator Experiments #1 and #2

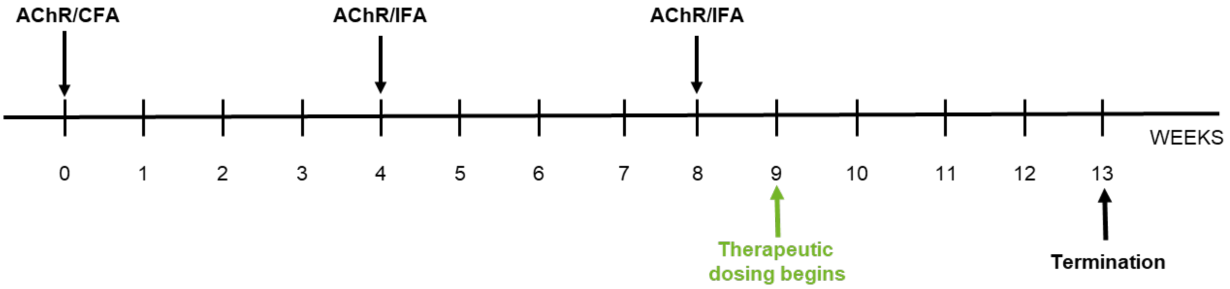

Comparator Experiment #1 Groups (all i.p. dosing):

1. PBS (n = 12); twice weekly from Week 9 to 12.
2. Povetacicept (~10 mg/kg; n = 12); twice weekly from Week 9 to 12.
3. Telitacicept (~12 mg/kg; n = 12); twice weekly from Week 9 to 12.
4. Fc Control (~8.3 mg/kg; n = 12); twice weekly from Week 9 to 12.

Comparator Experiment #2 Groups (all i.p. dosing):

1. PBS (n = 12); twice weekly from Week 9 to 12.5.
2. Povetacicept (~10 mg/kg; n = 12); twice weekly from Week 9 to 12.5.
3. Efgartigimod (~20 mg/kg; n = 12); twice weekly from Week 9 to 12.5.
4. Anti-CD20 antibody (0.25 mg; n = 12); once weekly from Week 9 to 12.
5. Fc Control (~8.3 mg/kg; n = 12); twice weekly from Week 9 to 12.5.

Supplementary Figure 1: Shown are the study designs and dosing regimens for each of the 3 experiments in which active EAMG was induced in female C57BL/6 mice as described in the Methods section. Injections with acetylcholine receptor (AChR) in either complete Freund’s adjuvant (CFA) or incomplete Freund’s adjuvant (IFA) were given subcutaneously every 4 weeks as shown. Each animal was weighed and scored at the beginning of each experiment, twice a week until the second boost (Week 4), and then every other day (or twice a day if the animal demonstrated severe weakness) by blinded investigators, unaware of the treatments, until termination 13 weeks following the initial immunization. See the Materials and Methods section for additional details.

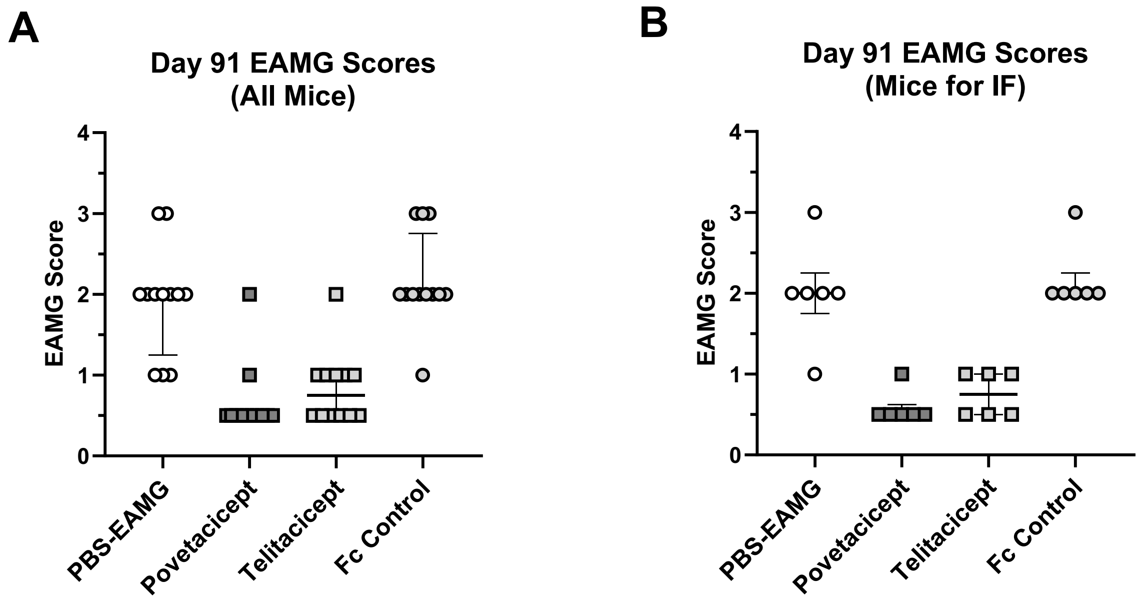

Supplementary Figure 2. EAMG scores at termination (Week 13) for **(A)** all mice (n = 12/group) and **(B)** for the subset of mice (n = 6/group) selected at random for immunofluorescence (IF) analysis of draining lymph nodes. Individual mice are plotted, with horizontal and vertical bars as the median and interquartile range, respectively. EAMG scores for each treatment group between A and B were not statistically significantly different from each other ($p = 0.95$ to >0.99 by Mann-Whitney test).

Supplementary Figure 3A

A

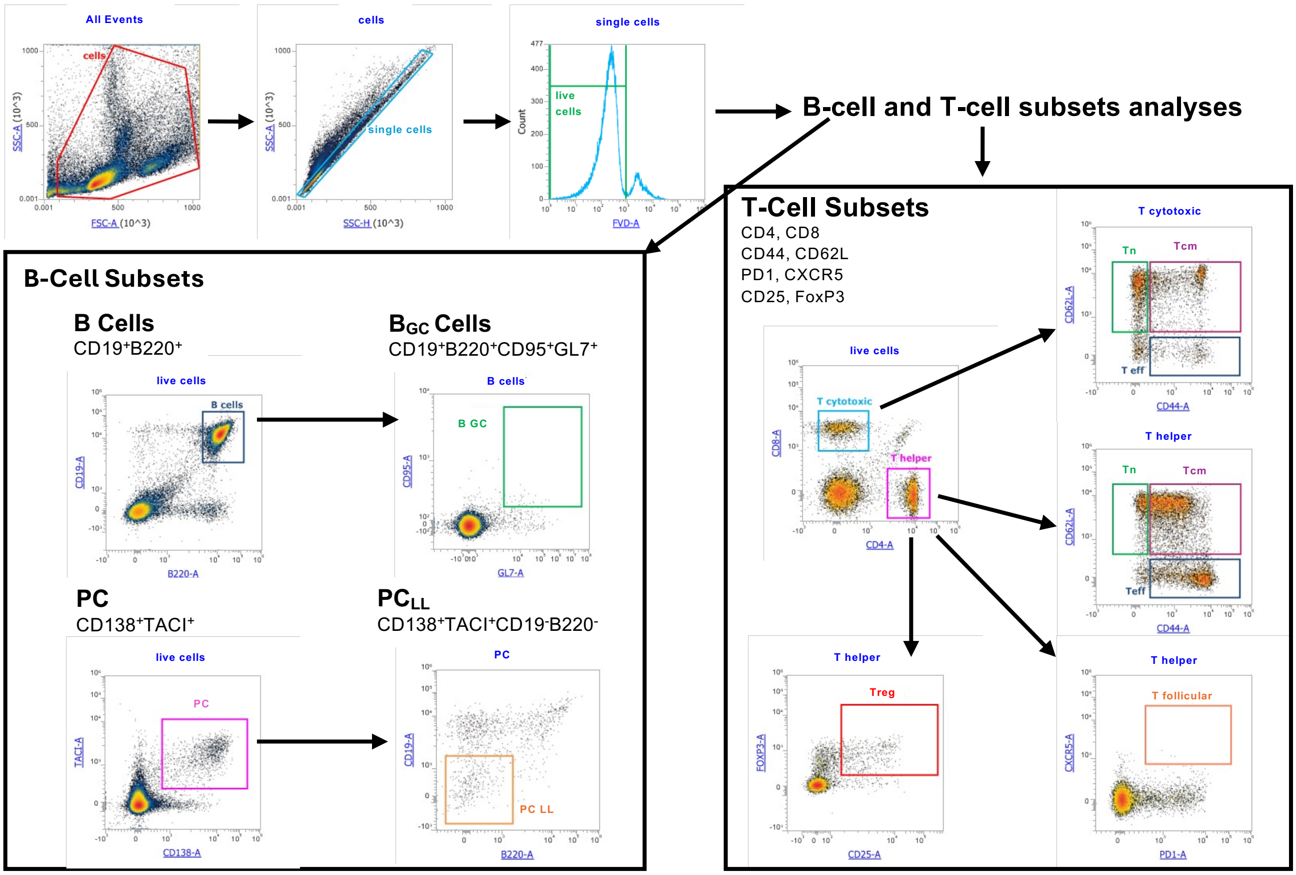

Supplementary Figure 3. Flow cytometry gating schemes are shown using cell population definitions as defined in the Methods. **(A)** Overall gating scheme detailing the identification of live single cells using side scatter area (SSC-A) and side scatter height (SSC-H) for single cells, followed by viability dye exclusion for live cells. The live cell gate was used as the basis for the identification and subsetting of B cells, plasma cells (PC), and T cells, using the cell markers as shown. Representative flow plots for each of the groups in comparator experiment #1 are shown for CD19+B220+ cells **(B)**, plasma cells **(C)**, and CD4+ and CD8+ T cells **(D)**. B_{GC} = germinal center B cells; PC_{LL} = long-lived plasma cells; Tn = naïve T cells; Tcm = central memory T cells; Treg = regulatory T cells; HD = healthy donor (naïve) mice.

Supplementary Figure 3B-E

B

CD19⁺B220⁺ Cells

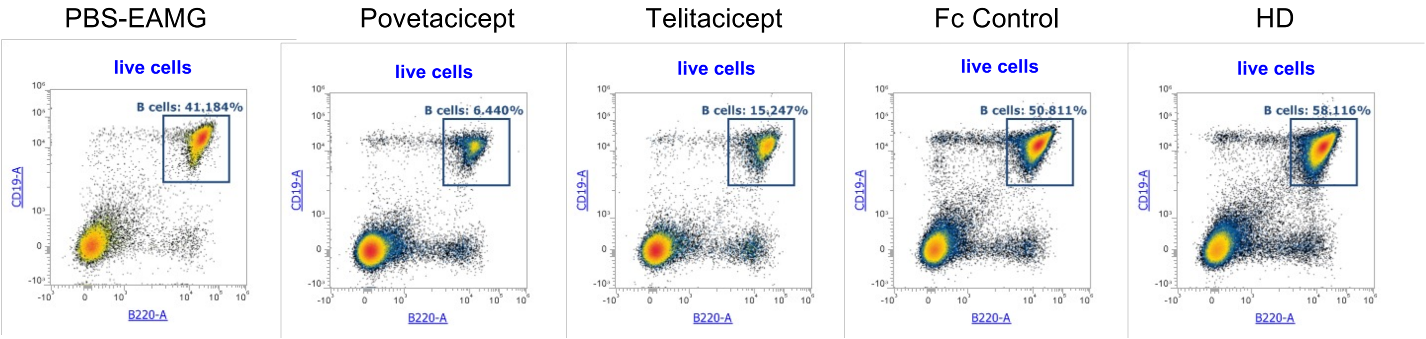

C

PC TACI+CD138+

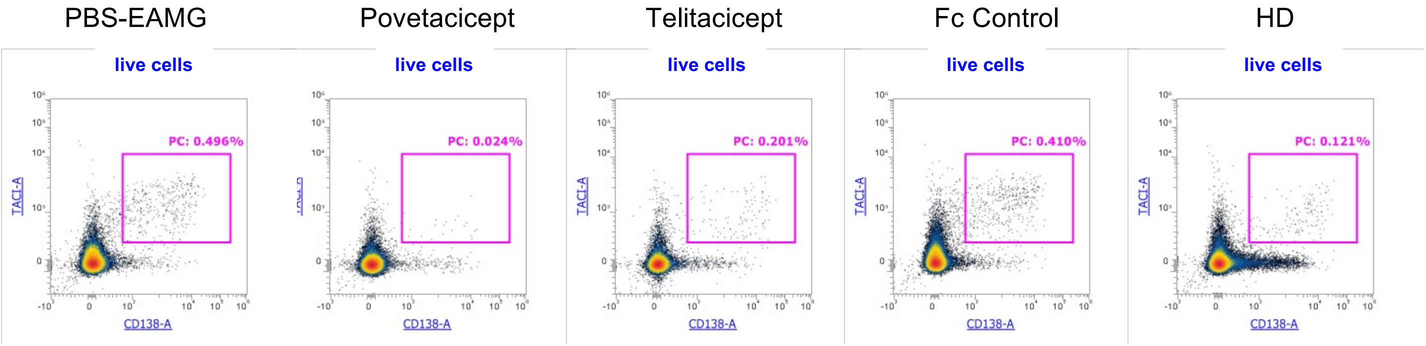

D

PC_{LL} TACI+CD138+CD19-B220-

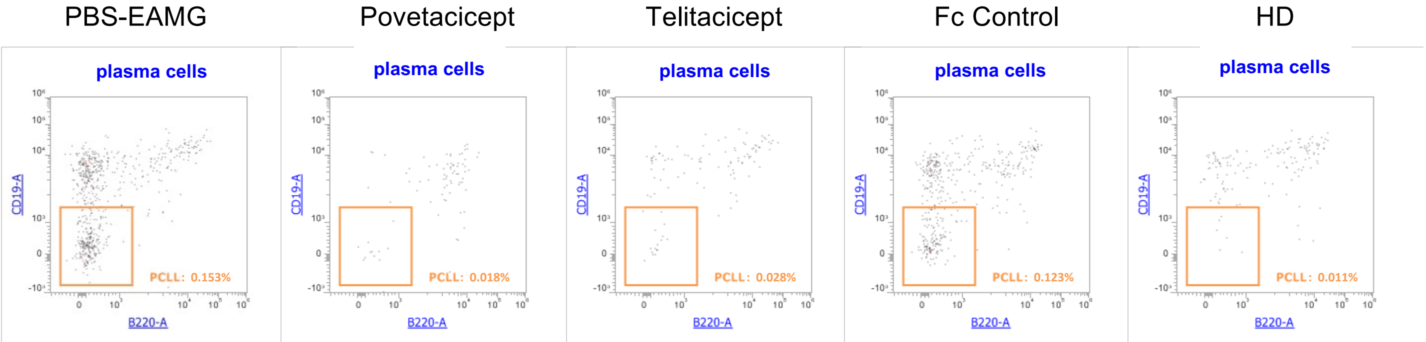

E

CD4⁺ and CD8⁺ T Cells

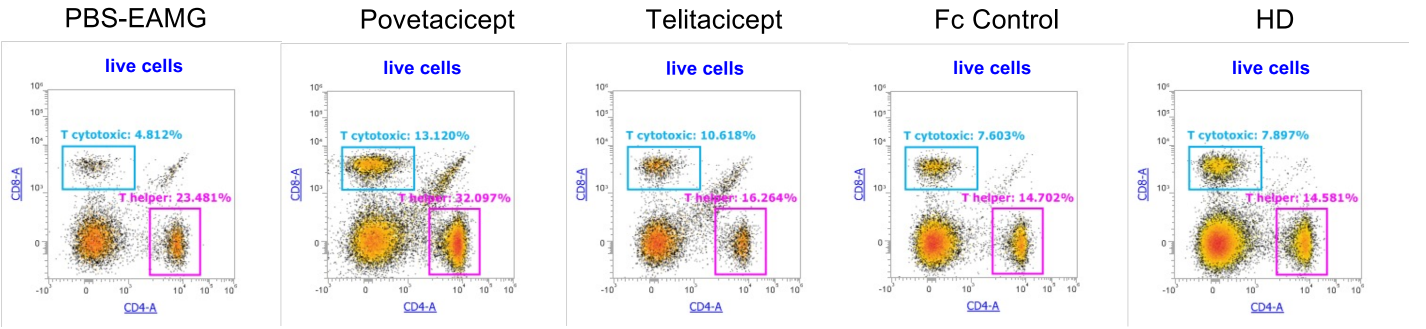

eTable 1. Antibodies used for flow cytometry analysis.

		Target	Fluorochrome	Dilution	Manufacturer	Catalog #	Clone #
**Spleen Flow cytometry**	*B cell panel*	CD138	Brilliant Violet 421 (BV421)	1:20	BioLegend	142507/23/08	281-2
		CD267 (TACI)	APC	1:80	Invitrogen eBioscience	17-5942-82	ebio8F10-3
		B220 (CD45R)	PE	1:160	BioLegend	103207/08	RA3-6B2
		CD19	PE-Cyanine7	1:300	BioLegend	115519/20	6D5
		FAS (CD95)	FITC	1:400	BioLegend	152605/06	SA367H8
		GL-7	PerCP-eFluor710	1:40	Invitrogen eBioscience	46-5902-82	GL7
	T cell panel	CD4	PerCP-Cyanine5.5	1:80	BioLegend	100433/434	GK1.5
		CD8	Pacific Orange	1:100	Invitrogen eBioscience	MDC0830	5H10
		CD62L	PE	1:160	BioLegend	104407/08	MEL-14
		CD44	NovaFluor Blue 610-70S	1:16	Invitrogen eBioscience	M010T02B06	IM7
		CD185 (CXCR5)	FITC	1:100	Invitrogen eBioscience	11-7185-82	SPRCL5
		CD279 (PD-1)	Brilliant Violet 421 (BV421)	1:160	BioLegend	135217/21/18	29F.1A12
		CD25	PE-Cyanine7	1:400	Invitrogen eBioscience	25-0251-82	PC61.5
		Foxp3	APC	1:20	Invitrogen eBioscience	17-5773-82	FJK-16s
**Lymph Node Imaging**		B220 (CD45R)	purified, primary mAb	1:100	Invitrogen eBioscience	14-0452-82	RA3-6B2
		secondary pAb	Goat anti-rat IgG, Alexa Fluor 555	1:1000	Invitrogen eBioscience	A-21434	
		Ki67	purified, primary mAb	1:200	Invitrogen eBioscience	MA5-14520	SP6
		secondary mAb	Goat anti-rabbit IgG, Alexa Fluor 488	1:1000	Invitrogen eBioscience	A-32731	
